# A narrative review of HIV surveillance strategies used in selected sub-Saharan African countries

**DOI:** 10.3389/fpubh.2026.1887244

**Published:** 2026-10-27

**Authors:** Marriott Nliwasa, Beth A. Tippett Barr, Fumbani Chigawa, Michael G. Chipeta, McEwen Khundi, Themba Mzembe, Brewster Moyo, Chikondi Mafaiti, Takondwa Msosa, Jeffrey W. Imai-Eaton, Victor Mwapasa

**Affiliations:** 1 School of Medicine and Oral Health, Kamuzu University of Health Sciences, Blantyre, Malawi; 2 Division of Epidemiology and Biostatistics, School of Public Health, Faculty of Health Sciences, University of the Witwatersrand, Johannesburg, South Africa; 3 School of Global and Public Health, Kamuzu University of Health Sciences, Blantyre, Malawi; 4 Nyanja Health Research Institute, Salima, Malawi; 5 African Institute for Development Policy, Lilongwe, Malawi; 6 Center for Communicable Disease Dynamics, Department of Epidemiology, Harvard T.H. Chan School of Public Health, Boston, MA, United States; 7 MRC Centre for Global Infectious Disease Analysis, School of Public Health, Imperial College London, London, United Kingdom

**Keywords:** Ghana, HIV, Kenya, Lesotho, Malawi, surveillance, Zimbabwe

## Abstract

**Background:**

Over the last two decades, new HIV infections and AIDS-related deaths have declined significantly through expansion of access to HIV testing, treatment, and prevention services. Data to monitor epidemic trends and program coverage have been central to driving progress. We describe HIV surveillance indicators and methods that countries use to monitor the HIV epidemic and impact of programs in selected African countries.

**Methods:**

We conducted a review of global and national HIV surveillance guidelines. We extracted information on (1) indicators used to monitor HIV epidemic changes, and (2) activities countries conduct to measure these. We conducted an in-depth document analysis for five selected African countries: Ghana, Kenya, Lesotho, Malawi, and Zimbabwe.

**Results:**

We identified 66 documents that inform HIV surveillance plans at national or global levels. Documents from the five selected countries reflected a range of 80 to 103 HIV indicators per country compared to 128 from a consolidated list of indicators in global guidance documents. The largest proportion of reported national indicators was for integrated services and non-biomedical measures such as HIV stigma and knowledge, with these accounting for a range of 22.8% (21/92) in Ghana to 64.1% (66/103) in Lesotho. Measuring incidence and mortality relied predominantly on mathematical modeling. Activities such as community and facility-based mortality surveillance, HIV drug resistance surveys and bio-behavioral surveys were specified in strategic plans but were partially implemented or not documented.

**Conclusion:**

HIV surveillance among selected African countries primarily used mathematical models for estimating HIV incidence and mortality and heavily depended on infrequent household surveys for quantifying epidemic burden. Strategy documents named many HIV indicators and surveillance activities, which were inconsistently implemented. Countries could reassess suitability of current indicators and activities and streamline these for efficient estimation of HIV burden.

## Introduction

The past two decades have seen substantial progress in reducing new HIV infections and AIDS-related deaths in Africa. From 2010 to 2024, the estimated number of new HIV infections in the UNAIDS West and Central region plus the Eastern and Southern region has declined from 1.44 million to 0.65 million per year, and the number of HIV-related deaths has declined from 0.91 million to 0.38 million (1, 2). These successes were attained through major efforts to provide near-universal access to HIV testing, care, treatment, and prevention services (3). Despite this success, new infections and deaths remain far above the globally adopted targets of 150,000 infections and 100,000 deaths per year for the region, threatening the achievement of the goal of ending HIV as a public health threat by 2030 in the region (4).

Since the onset of the epidemic, effective responses have relied on surveillance to quantify and project the epidemic (5), guide program strategies, and monitor impacts. Approaches have evolved substantially over the decades. Following the emergence of HIV in the early 1980s, countries instituted AIDS case reporting systems using case definitions, even before laboratory tests were available. When testing became available, efforts focused on unlinked anonymous surveys (6) and tracking of prevalence among sentinel populations (7), including pregnant women attending antenatal care (ANC) and specific at-risk populations such as sexually transmitted infections (STI) clinic clients, female sex workers, and blood donors (8). In 2000, the ‘second-generation’ HIV surveillance guidance released by WHO (updated in 2013) recommended country-tailored approaches that linked epidemiologic outcomes with behavioral risk factors and determinants of infection, including focus on areas such as HIV and AIDS case and mortality reporting, behavioral and bio-behavioral surveys, sentinel surveillance, surveillance for sexually transmitted infections (STIs), and size estimation of key populations (8). In high-prevalence settings, including most of Africa, second-generation surveillance also recommended national household surveys with serological testing to robustly measure adult HIV prevalence. By the mid-2000’s, Demographic and Health Surveys (DHS) replaced ANC data as the primary data for quantifying the magnitude of the epidemic in the general population. The 2010s saw the introduction of HIV-specific population surveys expanded to include biological markers such as detectable antiretrovirals, viral load, and antibody biomarkers for recent HIV infection, improving the ability to associate program interventions with population-level impact (9). There was also increased reliance on routine service delivery monitoring and evaluation data for epidemic monitoring, and efforts focused on scaling up electronic case reporting at individual and aggregate levels (10, 11).

Surveillance approaches and monitoring indicators have also evolved in response to emerging policy objectives and goals. UNAIDS’ 90–90-90 and subsequently 95–95-95 targets intended to support progress toward HIV epidemic control and ending AIDS as a public health threat by 2030 (12). Furthermore, a definition of HIV epidemic control is a scenario where the incidence, prevalence, morbidity, and mortality of HIV are reduced to a locally acceptable level through deliberate efforts, with continued interventions required (4). Experts have proposed a number of metrics to help track progress toward HIV epidemic control (4, 13), including: (1) Proportional annual reduction in new infections and deaths, (2) HIV incidence, (3) AIDS-related mortality, (4) Incidence-to-all-cause mortality ratio, and (5) Incidence-to-prevalence ratio. A key tenet of disease control is the surveillance required to demonstrate achievement and maintenance of disease control (4, 14).

During the rapid scale-up of prevention and treatment interventions over the past two decades, HIV surveillance methods were designed to measure large changes in intervention coverage and their impact on epidemic trends over long time intervals. However, as countries achieve the 95–95-95 targets (15) and seek to monitor lower levels and continued declines in new infections and AIDS-related deaths, changes in the epidemic trajectory become smaller and harder to monitor with precision and timeliness. This new era again necessitates shifts in approaches to epidemic monitoring. Here we reviewed relevant literature to assess current surveillance approaches and indicators that selected African countries use to monitor the trajectory of HIV epidemic. We also assessed whether activities stated in surveillance strategies were actually implemented by the countries. Therefore, this review compares surveillance activities prioritized in national strategies with available evidence of their implementation in the five selected countries.

## Methods

### Document search

We conducted a narrative review of grey literature focusing on national and global or regional guidance documents on HIV surveillance, published between 2014–2025. We included global and regional documents to compare with national strategy documents. We searched in document repositories of HIV multinational organizations and national HIV-specific government departments such as National AIDS Commissions. The search was conducted via the Google search engine to access relevant documents for each country; we repeated the following search for each country: *[Country] AND HIV/AIDS AND (surveillance OR Monitoring OR Evaluation OR Strategy OR Framework OR Guideline OR Plan OR PEPFAR OR CDC OR “Global Fund”)*. Additionally, we purposively searched for HIV/AIDS surveillance guidelines or strategies from UNAIDS, WHO and PEPFAR. The search process was iterative and considered complete per country once national HIV strategy documents covering the period from 2014 to 2025 were identified. A list of documents identified is presented in Supplementary Tables S1, S2. Further analysis focused on the most recent strategy documents from each country; superseded documents were excluded.

### Document review and data abstraction

Two researchers (MN and BTB) identified relevant global, regional, and national documents published in English and considered them for inclusion if they covered HIV indicators or HIV surveillance methods and were published since the launch of the UNAIDS 90–90-90 targets in 2014. For in-depth analysis, we purposively selected documents from five African countries: West Africa (Ghana), East Africa (Kenya), and Southern Africa (Lesotho, Malawi, and Zimbabwe). We selected these countries for ease of access to stakeholders to confirm implementation activities.

We extracted indicators from the selected documents by referencing indicator lists attached in appendices of the documents and identifying any additional indicators referenced in the document text. One reviewer extracted indicators from the documents; the extracted list was shared with the study team for review. HIV indicators were classified into four categories of domains compiled from the WHO, UNAIDS, and PEPFAR monitoring frameworks: (1) HIV services, each further divided into logic framework categories (inputs, activities, outputs, outcomes and impacts); (2) epidemiological indicators of morbidity (incidence and prevalence) and mortality; (3) HIV risk (such as occurrence of STIs); and (4) Non-biomedical and integrated services (such as stigma and comorbidity screening) (Figure 1).

**Figure 1:**
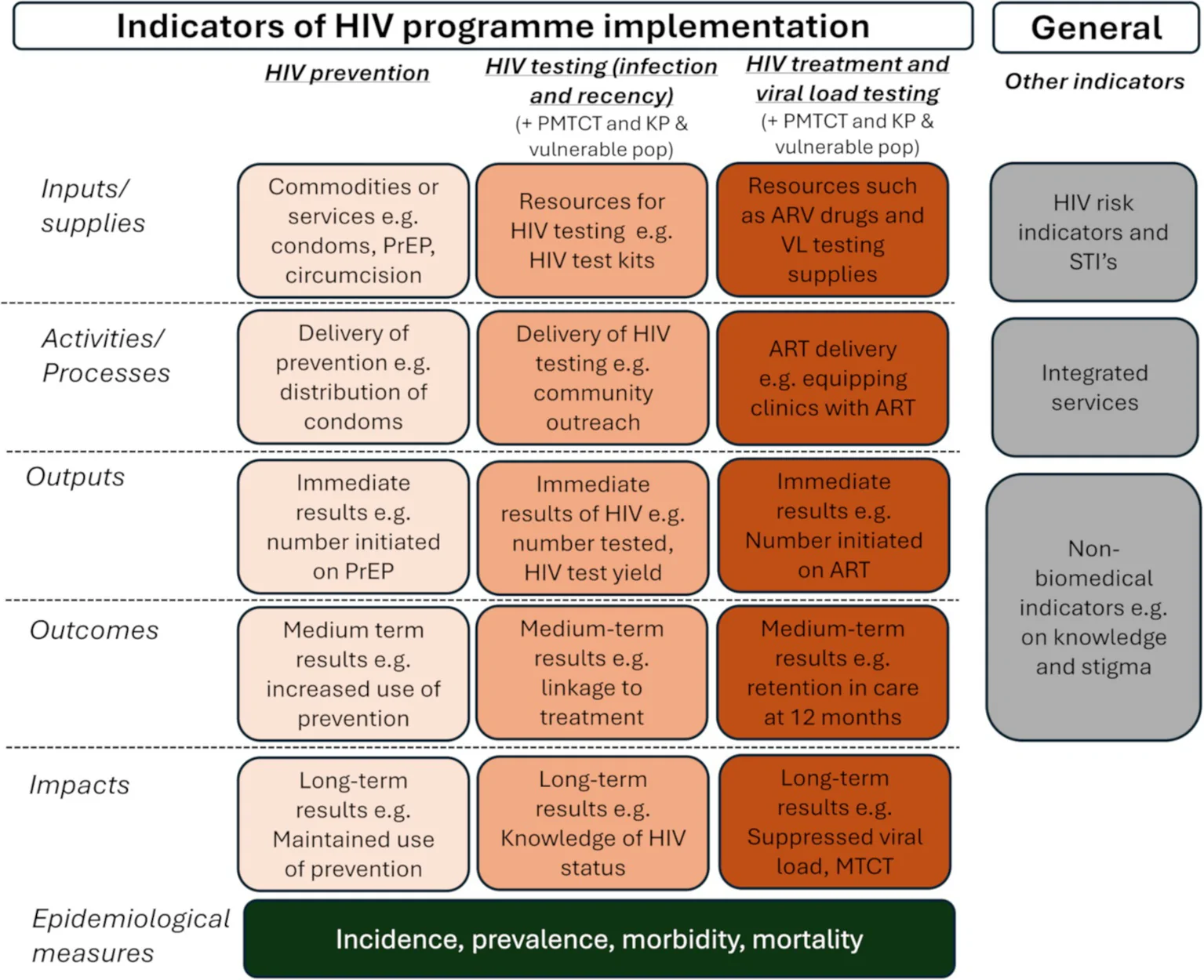
Framework for classification of surveillance indicators. PrEP, pre-exposure prophylaxis; ART, antiretroviral therapy; ARV, antiretroviral drugs; MTCT, mother to child transmission; STI, sexually transmitted infections.

### Description of HIV surveillance approaches in focus countries

We used a framework from Diaz et al. to assess surveillance methods in each country (16). Briefly, this involved assessing whether a country conducts activities such as surveys, sentinel HIV surveillance, and routine facility-based data collection and analysis aimed at gathering information on HIV risk and trends in HIV incidence, morbidity, and mortality (Figure 2). For each country, we first reviewed documents to identify planned surveillance activities. Second, we identified whether the documented surveillance activities were fully or partially implemented or not documented. We also documented the periodicity and recency of implementation of the surveillance approaches, including whether activities were conducted within the last 5 years. The full extracted tables for the selected countries are presented in Supplementary Tables S4–S8. To ascertain whether surveillance activities were conducted in the selected countries, we looked for reports, for example, survey reports. In addition, we shared the list of the identified surveillance activities for each country with monitoring and evaluation officers of the HIV and AIDS commissions of the selected countries, or other stakeholders such as those from Centers for Disease Control (one person per country), for confirmation of the classification of whether the activity was done in the country and if it was done within the last 5 years. To facilitate the consultation process, we shared Excel sheets with stakeholders containing extracted information per country, including our initial classification. During stakeholder validation, stakeholders confirmed whether each classification was correct. There were no changes to the initial classification.

**Figure 2:**
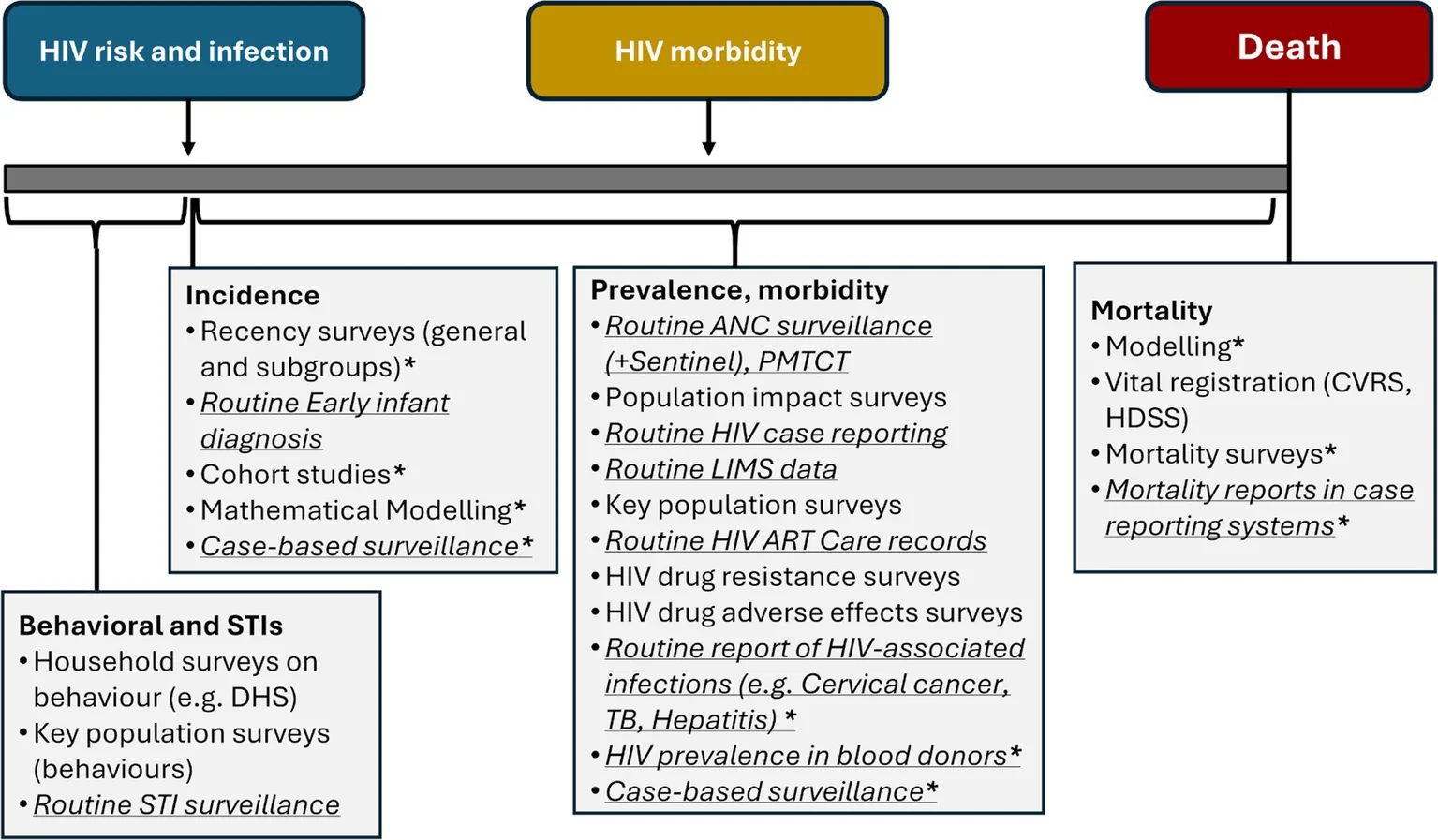
Common approaches for HIV surveillance in high burden countries. Adapted from Diaz et al. (16), asterisks show items added to the framework. Underlined and italicized approaches refer to *routine health facility data sources*. DHS, Demographic and Health Survey; MICS, Multiple Indicator Survey; STI, Sexually Transmitted Infections; AGYW, Adolescent Girls and Young Women; ANC, Antenatal Care; ART, Antiretroviral Therapy; PMTCT, Prevention of Mother to Child Transmission; LIMS, Laboratory Information Management System; NHLS, National Health Laboratory System; CVRS, Civil Registration and Vital Statistics; HDSS, Health and Demographic Surveillance System.

## Results

### Characteristics of included documents

We screened 122 national-level documents and included 45 documents from 22 countries in our high-level review and five in our in-depth review (Figure 3). These documents were primarily national HIV strategic plans, HIV clinical care guidelines, and PEPFAR Country Operational Plans (COPs). At the global level, we screened 43 documents, of which 21 were included for analysis, from UNAIDS and WHO (*n* = 10), the Global Fund (*n* = 6), PEPFAR (*n* = 3), the African Union (*n* = 1), and the European Centre for Disease Prevention and Control (*n* = 1).

**Figure 3:**
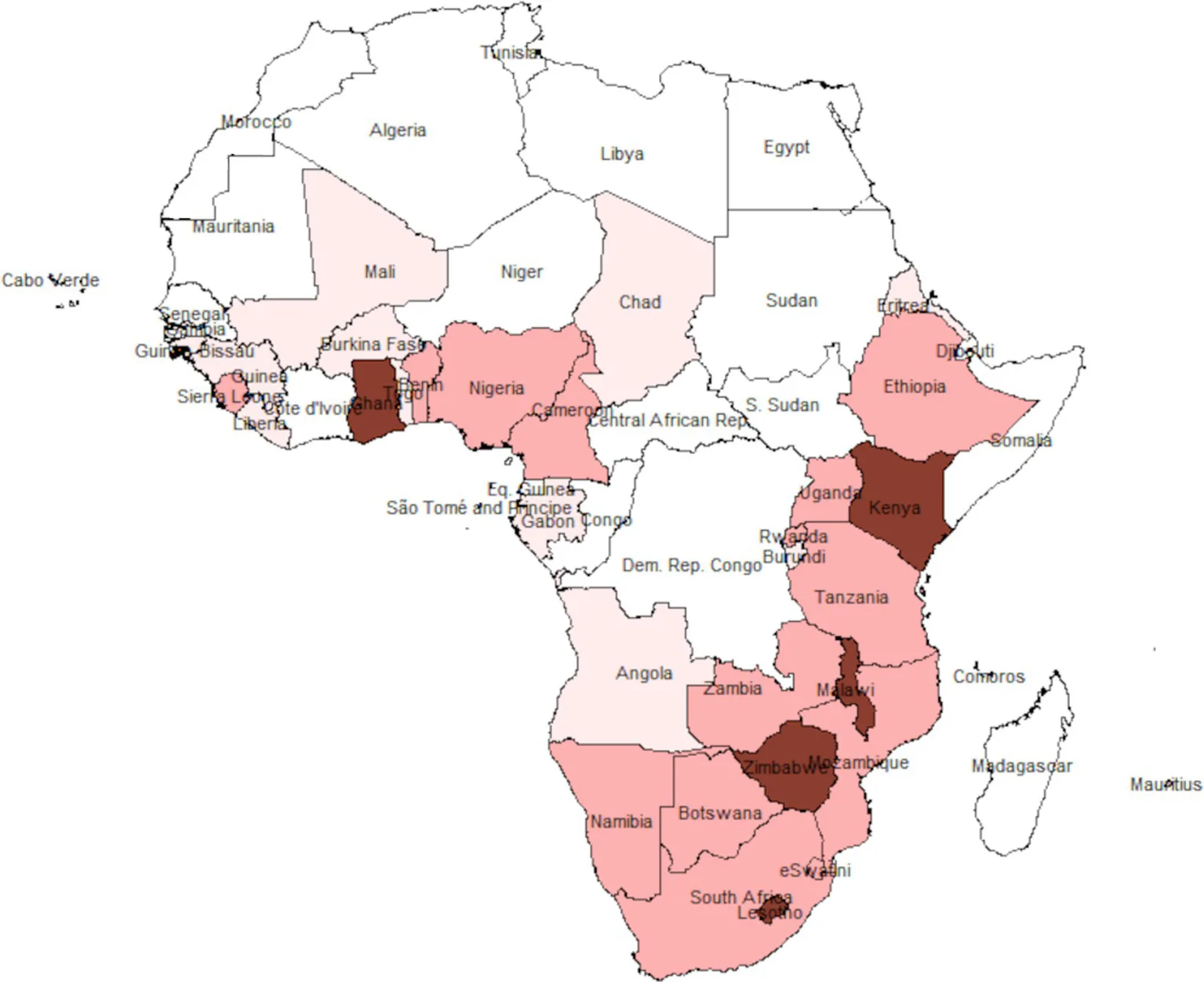
Map showing countries with included documents.

### Country-level HIV indicators

HIV strategic documents from the five focus countries (Ghana, Kenya, Lesotho, Malawi and Zimbabwe) contained a range of 80 to 103 indicators (Figure 4) (17–21). The country documents had varying emphasis on specific areas (Figure 4), with the largest proportion of reported national indicators being classified as non-biomedical or integrated services, ranging from 22.8% (21/92) in Ghana to 64.1% (66/103) in Lesotho (Figure 4). The number of extracted indicators per country was lower than the number in a consolidated list of indicators from global HIV monitoring guidance documents by UNAIDS, Global Fund and PEPFAR (Figure 4). A comparison of indicators extracted from documents of focus countries and indicators from international agencies was done for HIV focused epidemiological indicators, HIV service outputs, outcomes and impact indicators and HIV risk indicators. For example, in the Zimbabwe documents, we extracted 7 of 8 international HIV-focused epidemiological indicators; 23 of 27 international HIV service outputs, outcomes and impact indicators; and 1 of 7 international HIV risk indicators.

**Figure 4:**
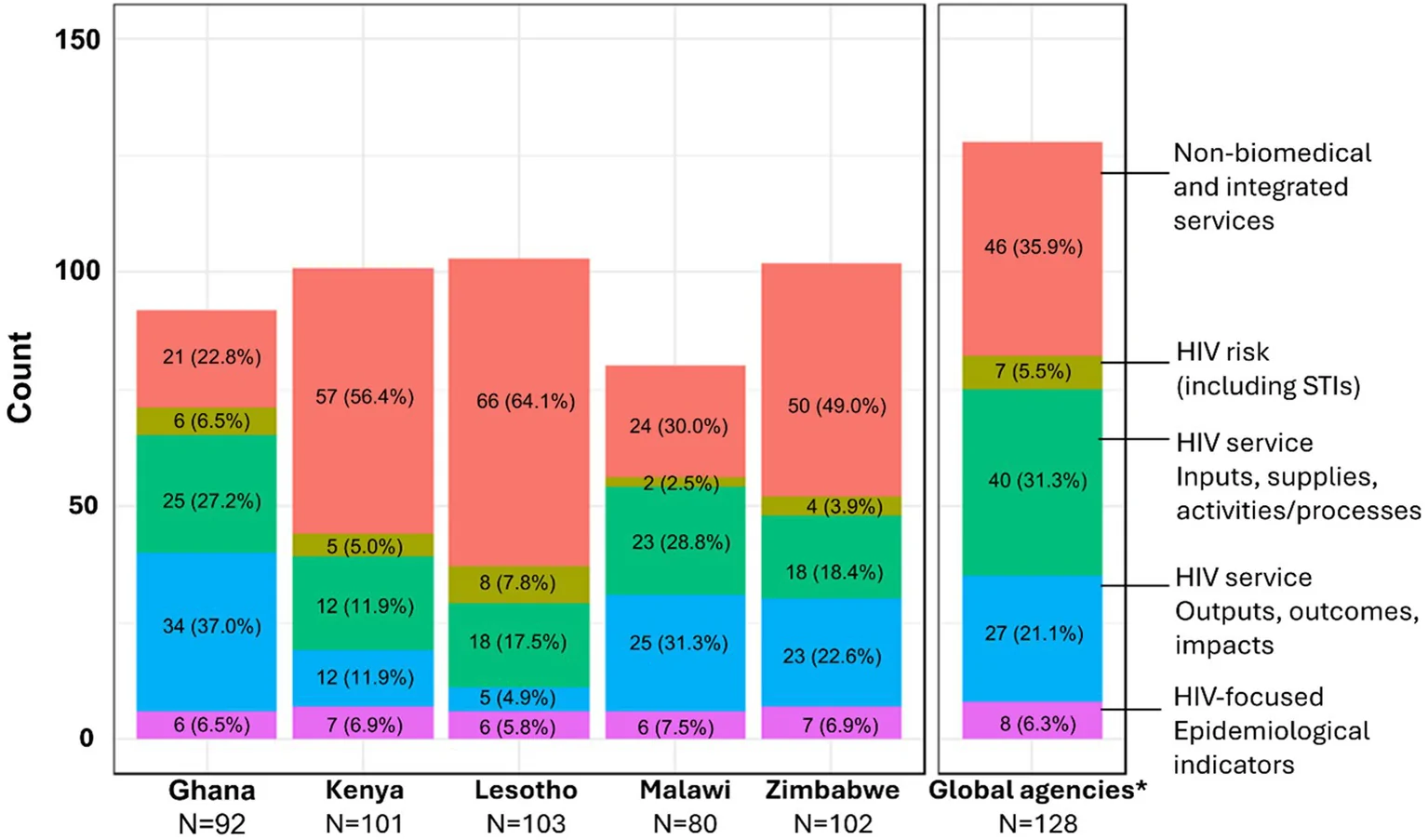
Type of HIV indicators by country. *Refers to the consolidated list of indicators by UNAIDS, Global Fund and PEPFAR available in Supplementary materials.

### HIV indicator measurement approaches and implementation

Figure 5 presents information on surveillance approaches used in the focus countries. To measure HIV incidence, documents predominantly relied on outputs of mathematical modeling (Figure 5). Kenya and Zimbabwe had localized cohort studies as additional data sources for incidence (18, 21–23). National HIV incidence was also empirically measured through PEPFAR-funded Population HIV Impact Assessment (PHIA) surveys conducted between 2015 and 2022. Among the five countries, national PHIAs had been conducted twice in the last seven years in Malawi (2015/16 and 2020/21), Zimbabwe (2015/16 and in 2020), and Lesotho (2016/17 and 2020) (24). PHIA was conducted once in Kenya, and HIV incidence was measured (25). Ghana had never measured incidence in national household surveys. However, sustainability of quinquennial PHIA implementation was noted as a concern; for example, the Zimbabwe National HIV and AIDS Strategic Plan, 2021–2025 stated: “there is no clear strategy for resource mobilization for surveys” (21). All countries’ documents reported using modeling for estimating HIV-related mortality. Kenya had additional facility-based (26) and community-based HIV mortality surveillance mechanisms. Community-level mortality surveillance was through well-established Health and Demographic Surveillance Sites (HDSS) (27). Kenya also conducted specific surveys for assessing stigma and discrimination against PLHIV (28). All five focus countries had incorporated measurement of key behavioral factors affecting HIV risk in their population-based household surveys.

**Figure 5:**
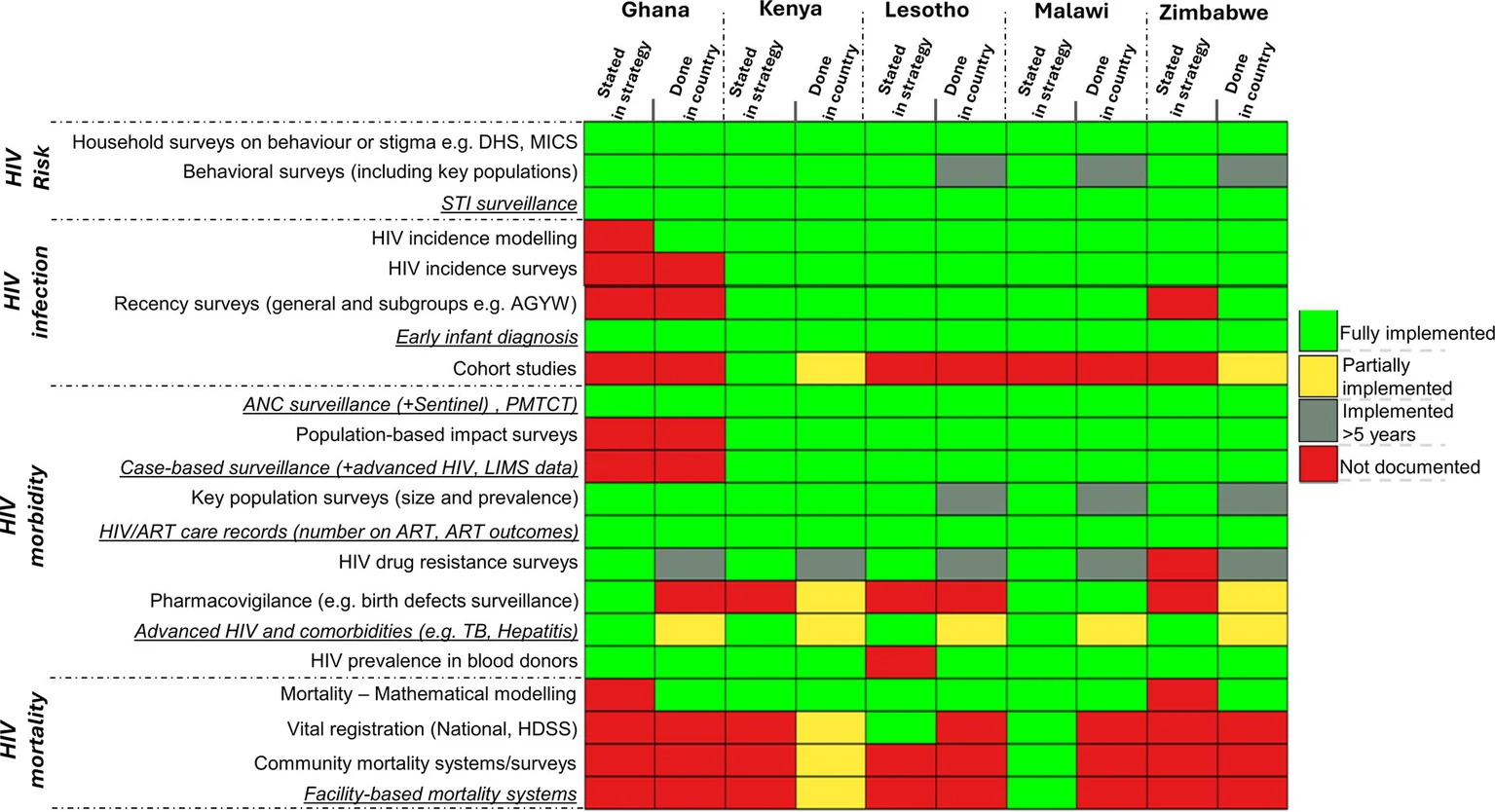
Assessment of available HIV surveillance approaches in selected African countries. Underlined and italicized approaches refer to routine health facility data sources. Partial is when activity not fully implemented or implemented at subnational level. DHS, Demographic and Health Survey; MICS, Multiple Indicator Survey; STI, Sexually Transmitted Infections; AGYW, Adolescent Girls and Young Women; ANC, Antenatal Clinic; PMTCT, Prevention of Mother to Child Transmission; LIMS, Laboratory Information Management System; HDSS, Health and Demographic Surveillance Site. Strategy refers to national government HIV strategy.

Discrepancies existed between the surveillance activities described in national HIV strategic plans and what was implemented (Figure 5). For example, in Malawi, the national HIV strategic plan mentioned the use of various methods for HIV mortality measurement, including vital registration and community- and facility-based mortality surveillance, but there was no documented evidence of implementation at national level (20). Additionally, none of the five focus countries had conducted drug resistance surveys in the last five years, as specified in surveillance plans, and biobehavioral surveys (BBSSs) in key populations had been conducted only in Malawi and Kenya in the last five years (Figure 5).

## Discussion

In this narrative review, we found that HIV surveillance among the selected African countries is characterized by reliance on mathematical models as the primary source for epidemiological outcomes for HIV incidence and mortality, use of routine program data to track impacts of service delivery, and heavy dependence on infrequent household surveys for quantifying epidemic burden and behavioral risk indicators. There were inconsistencies in surveillance methods prioritized in national strategic plans compared to what was implemented, with some countries implementing surveillance activities not described in their national strategic plans, while in others, planned surveillance activities were delayed or not documented, raising concerns that data are not available on the anticipated schedule to inform updates to national strategic plans. We consistently extracted fewer indicators in individual countries than in a consolidated list of international guidance documents. We found that countries linked to international agencies were probably measuring these indicators, but they had not included them in national strategy documents as key indicators. This reveals a possible disconnect between country prioritization processes and international requirements.

In the last twenty years, HIV programs have relied heavily on 5-yearly population-based surveys, interpreted and extrapolated through mathematical modeling, to monitor the epidemic (9). However, their infrequency results in limited timely data on national HIV trends to estimate incidence, and no data on subnational incidence, which is key to monitoring the epidemic trajectory. National strategy documents and surveillance implementation both described innovations in monitoring incidence and new infections using case-based surveillance and routine recent infection testing among new diagnoses. The use of routine health facility-based data may provide real-time information on the state of the HIV epidemic. However, it is important to address limitations of routine data such as selection bias, incomplete linkage across testing and treatment platforms and duplicate records (29). There is a need to develop mathematical models that use routine data and inform surveillance indicators such as incidence, while accounting for selection biases associated with differences in health care access and coverage. Programs may consider sentinel sites with high-quality routine facility-based data collection to ensure adequate surveillance data during a period of overall health data system strengthening.

Measuring HIV-related mortality is a major challenge in the selected African countries. HIV mortality estimates are predominantly based on mathematical modeling, given the lack of reliable vital registration systems in the selected countries, especially for PLHIV mortality. Kenya had some evidence of improving their mortality surveillance in pockets of high HIV-burden areas (30). Systematic analysis of trends in numbers of people detected with advanced HIV and related conditions is an important measure of HIV morbidity and a potential proxy for mortality trends (27, 31, 32). One innovative method was used in Malawi, where in-hospital admissions and deaths of people living with HIV were expressed relative to general population sizes to determine changes in rates of HIV positive admission over time (32).

This study had some limitations. The review mainly focused on national-level guidance documents and may have missed subnational-level surveillance activities being implemented by non-governmental agencies. Other limitations include restriction to English-language documents; possible omission of unpublished or internal program documents; and limited representativeness of the five focus countries.

## Conclusion

With the changing trajectories of both the HIV epidemic and potential challenges in availability of financial resources for various surveillance activities in selected African countries, there is a need for countries to review the focus and sustainability of their HIV surveillance methods. HIV incidence, prevalence and mortality remain key indicators for tracking nations’ progress toward achievement of the goal of ending AIDS as a public health threat. Ensuring continuous measurement of HIV incidence requires innovation in methodological approaches to quantifying incidence using routine health facility-based data together with targeted, efficient surveys. Countries should prioritize fewer but more frequent decision-relevant indicators, improved routine data quality, targeted surveys, and better mortality surveillance through stronger civil registration and vital statistics systems.

## Glossary

AGYW: Adolescent Girls and Young Women
ANC: Antenatal Care
AIDS: Acquired Immunodeficiency Syndrome
ART: Antiretroviral Therapy
ARV: Antiretroviral Drugs
CDC: Centers for Disease Control and Prevention
CRVS: Civil Registration and Vital Statistics
DHS: Demographic and Health Survey
HDSS: Health and Demographic Surveillance Site/System
HIV: Human Immunodeficiency Virus
LIMS: Laboratory Information Management System
MICS: Multiple Indicator Cluster Survey
MTCT: Mother-to-Child Transmission
NHLS: National Health Laboratory System
PEPFAR: President’s Emergency Plan for AIDS Relief
PHIA: Population-based HIV Impact Assessment Survey
PLHIV: People Living with HIV
PMTCT: Prevention of Mother-to-Child Transmission
PrEP: Pre-Exposure Prophylaxis
STI: Sexually Transmitted Infection
UNAIDS: Joint United Nations Programme on HIV/AIDS
WHO: World Health Organization
