# Supplementary material for: A narrative review of HIV surveillance strategies used in selected sub-Saharan African countries
Source: Front Public Health. 2026 Oct 27;14:1887244. doi: 10.3389/fpubh.2026.1887244 (PMC13748826; doi:10.3389/fpubh.2026.1887244)
Supplement: Supplementary file 1 [file Supplementary_file_1.DOCX]

**Table S1. Assessment of documents at country-level**

| **Document number** | **Country** | **Document title** | **Year of publication** | **Describes surveillance approach (Yes/No)** | **Indicators included**  **{Yes/No}** | **Include / Exclude** |
| --- | --- | --- | --- | --- | --- | --- |
| AGO_01 | Angola | National Strategy Plan on HIV/AIDS | 2008 | No | No | exclude |
| AGO_02 | Angola | Angola Country Operational Plan COP 2021 Strategic Direction Summary | 2021 | No | No | exclude |
| BEN_01 | Benin | 2022-2027 Benin Strategic Framework | 2022-2027 | yes | no | **include** |
| BWA_01 | Botswana | Botswana HIV Prevention Road Map 2023-2025 | 2023-2025 | yes | yes | **include** |
| BWA_02 | Botswana | COP 2021 Updates to Botswana’s Country Operational Plan 2020 Strategic Direction Summary | 2021 | No | No | exclude |
| BWA_03 | Botswana | 2021 Botswana HIV case surveillance implementation guide | 2021 | yes | no | **include** |
| BWA_04 | Botswana | National Strategic Plan to Reduce Human Rights – Related Barriers to HIV and TB Services: Botswana 2020 – 2025 | 2020 – 2025 | No | No | exclude |
| BWA_05 | Botswana | The Second Botswana National Strategic Framework for HIV and AIDS 2010–2016 | 2010–2016 | No | No | exclude |
| CMR_01 | Cameroon | cameroon-national-strategic-plan-for-fight-against-hivaids-and-stis-2021-2023 | 2021-2023 | yes | no | **include** |
| CAR_01 | Central Africa Republic | National_strategic_plan_for_the_fight_against_hiv_and_aids_in_the_central_african_republic_2023-2027 | 2023-2027 | maybe-mostly M&E | yes | **include** |
| COM_01 | Comoros | Plan strategique national (psn) de lutte contre le VIH/LE SIDA 2011-­‐2015 | 2011‐2015 | No | No | exclude |
| COD_01 | Democratic Republic of Congo | Democratic Republic of the Congo Country Operational Plan (COP) 2021 | 2021 | No | No | exclude |
| COD_02 | Democratic Republic of Congo | DRC-national-strategic-plan-against-HIV-2014-2017 | 2014-2017 | No | No | exclude |

**Continued on next page**

**Table S1. Assessment of documents at country-level (Continued)**

| **Document number** | **Country** | **Document title** | **year of publication** | **Describes surveillance approach (Yes/No)** | **Indicators included**  **{Yes/No}** | **include/exclude** |
| --- | --- | --- | --- | --- | --- | --- |
| ERI_01 | Eritrea | National Strategic Plan on HIV/AIDS/STIs 2003 -2007 | 2003 -2007 | old | old | exclude |
| ETH_01 | Ethiopia | HIV/AIDS National Strategic Plan (NSP) for Ethiopia 2021-2025 | 2021-2025 | yes | yes | **include** |
| SWZ_01 | Eswatini | Eswatini-COP22-Approval-Memo | 2022 | No | No | exclude |
| SWZ_02 | Eswatini | Eswatini Country Operational Plan (COP) 2023 | 2023 | yes | yes | **include** |
| GMB_01 | Gambia | National Policy Guidelines on HIV and AIDS 2014 - 2020 | 2014 - 2020 | yes | no | Exclude |
| GHA_01 | Ghana | National HIV AND AIDS, STI policy | 2013 | No | No | exclude |
| GHA_02 | Ghana | Consolidated guidelines for HIV care in Ghana | 2022 | No | No | exclude |
| GHA_03 | Ghana | National HIV & AIDS strategic plan 2021-2025 | 2021-2025 | maybe | yes | **include** |
| GHA_04 | Ghana | Ghana national HIV AND AIDS strategic plan 2016 - 2020 | 2016 - 2020 | No | No | exclude |
| GNB_01 | Guinea-Bissau | Plano estratégico nacional de luta contra vih/sida 2007 - 2011 ,guiné bissau | 2007 - 2011 | No | No | exclude |
| KEN_01 | Kenya | Kenya AIDS Strategic Framework II | 2020/21-2024/25 | no | yes | **include** |
| KEN_02 | Kenya | Kenya-COP22-Approval-Memo | 2022 | No | No | exclude |
| KEN_03 | Kenya | KENYA Country Operational Plan 2022 | 2022 | yes | yes | **include** |
| KEN_04 | Kenya | The national AIDS control council | 2021-2027 | no | no | exclude |
| LSO_01_ | Lesotho | The Lesotho program Working with Women, Children, and Families to End Pediatric AIDS | 2017 | no | no | exclude |
| LSO_02 | Lesotho | Policy framework on HIV/AIDS prevention, control and management | 2000 | no | no | exclude |
| LSO_05 | Lesotho | National HIV & AIDS Strategic Plan 2018/19 2022/23 | 2018/19 2022/23 | yes | yes | **include** |
| LSO_03 | Lesotho | Lesotho Country Operational Plan COP 2022 | 2002 | yes | yes | **include** |
| LSO_04 | Lesotho | Lesotho Country Operational Plan COP 2023 | 2023 | yes | yes | **include** |

**Continued on next page**

| **Document number** | **Country** | **Document title** | **year of publication** | **Describes surveillance approach (Yes/No)** | **Indicators included**  **{Yes/No}** | **include/exclude** |
| --- | --- | --- | --- | --- | --- | --- |
| LBR_01 | Liberia | National HIV/AIDS strategic framework II 2010‐2014 | 2010‐2014 | no | no | exclude |
| LBR_02 | Liberia | National HIV & AIDS strategic plan 2015 - 2020 | 2015 - 2020 | no | no | exclude |
| MWI_01 | Malawi | Country Operational Plan | 2023-2027 | yes | yes | **include** |
| MWI_02 | Malawi | COP 2021 | 2020-2025 | no | no | exclude |
| MWI_03 | Malawi | [National health indicators handbook for monitoring health sector performance](https://www.healthdatacollaborative.org/fileadmin/uploads/hdc/Documents/Country_documents/Malawi_National_Health_Indicators_FINAL_v11_clean_wt_sign_combo.pdf) | 2018 | yes | yes | **include** |
| MWI_04 | Malawi | [National HIV and STI Surveillance Strategic Plan: 2020-2025](https://mail.google.com/mail/u/0/?tab=rm&ogbl#inbox?projector=1&messagePartId=0.1) | 2020-2025 | yes | yes | **include** |
| MWI_05 | Malawi | National-Strategic-Plan-for-HIV-and-AIDS-2020-25-Final + unpublished surveillance document | 2020-2025 | yes | yes | **include** |
| MWI_06 | Malawi | Revised national HIV prevention strategy | 2018-2020 | maybe-mostly M&E | yes | **include** |
| MWI_07 | Malawi | National HIV and AIDS Policy 2022-2027 | 2022-2027 | yes | yes | **include** |
| MUS_01 | Mauritius | Republic of Mauritius national HIV action plan 2023 – 2027 | 2023 – 2027 | maybe-mostly M&E | yes | **include** |
| MOZ_01 | Mozambique | Mozambique Country Operational Plan 2023 | 2023 | yes | yes | **include** |
| MOZ_02 | Mozambique | HIV prevention perspective 2023 | 2023 | no | no | exclude |
| NAM_01 | Namibia | National Guidelines for Antiretroviral Therapy Fifth Edition 2016 | 2016 | no | no | exclude |
| NAM_02 | Namibia | National Strategic Framework for HIV and AIDS Response in Namibia 2017/18 to 2021/22 | 2017/18 to 2021/22 | no | no | exclude |
| NAM_03 | Namibia | Namibia Country Operational Plan (COP) 2021 | 2021 | no | no | exclude |
| NAM_04 | Namibia | National Strategic Framework for HIV and March 1, 2023 AIDS Response in Namibia | 2023/24 to 2027/28 | yes | yes | **include** |

**Table S1. Assessment of documents at country-level (Continued)**

**Continued on next page**

| **Document number** | **Country** | **Document title** | **year of publication** | **Describes surveillance approach (Yes/No)** | **Indicators included**  **{Yes/No}** | **include/exclude** |
| --- | --- | --- | --- | --- | --- | --- |
| NGA_01 | Nigeria | National HIV/AIDS Strategic Framework | 2017– 2021 | No | No | exclude |
| NGA_02 | Nigeria | NATIONAL HIV AND AIDS STRATEGIC FRAMEWORK 2021-2025 | 2021-2025 | maybe-mostly M&E | yes | **include** |
| RWA_01 | Rwanda | Rwanda HIV and AIDS National Strategic Plan 2013 – 2018 | 2013 – 2018 | old | old | exclude |
| RWA_02 | Rwanda | Rwanda HIV and AIDS National Strategic Plan 2013 – 2018, extension 2018-2020 | 2013 – 2018 | yes -but update available | yes-but update available | **include** |
| RWA_03 | Rwanda | National HIV/AIDS targets 2018-2020-2030 | 2018-2020-2030 | yes | yes | **include** |
| RWA_04 | Rwanda | Rwanda National Strategic Plan on HIV and AIDS 2009-2012 | 2009-2012 | old | old | exclude |
| RWA_05 | Rwanda | Rwanda National Strategic Plan on HIV and AIDS 2018-2024 | 2018-2024 | yes | yes | **include** |
| RWA_06 | Rwanda | Rwanda Country Operational Plan (COP/ROP) 2022 | 2022 | yes | yes | **include** |
| RWA_07 | Rwanda | Rwanda-National-HIV-Guidelines-for-Prevention-Management-of-HIV-2020 | 2020 | Update available | No | exclude |
| SLE_01 | Sierra Leone | HIV surveillance among fisherfolks in Sierra leone | 2011 | old | old | exclude |
| SLE_02 | Sierra Leone | Military surveillance protocol | 2003 | old | old | exclude |
| SLE_03 | Sierra Leone | Hiv surveillance on mine workers in sierra leone | 2008 | old | old | exclude |
| SLE_04 | Sierra Leone | National HIV AIDS Monitoring and Evaluation Framework 2006-2010 | 2006-2010 | old | old | exclude |
| SLE_05 | Sierra Leone | National M and E Manual Indicators | 2003 | old | old | exclude |
| SLE_06 | Sierra Leone | Protocol for hiv surveillance among the police force in sierra leone | 2003 | old | old | exclude |
| SLE_07 | Sierra Leone | National Monitoring & Evaluation Plan on HIVAIDS 2011-2015 | 2011-2015 | old | old | exclude |
| SLE_08 | Sierra Leone | National Strategic Plan on HIV/AIDS 2016-2020 | 2016-2020 | maybe-mostly M&E | yes | **include** |
| SLE_09 | Sierra Leone | Report on surveillance among the police in Sierra Leone | 2007 | old | old | exclude |
| SLE_10 | Sierra Leone | Surveillance among tuberculosis patients in Sierra Leone protocol | 2003 | old | old | exclude |

**Table S1. Assessment of documents at country-level (Continued)**

**Continued on next page**

| **Document number** | **Country** | **Document title** | **year of publication** | **Describes surveillance approach (Yes/No)** | **Indicators included**  **{Yes/No}** | **include/exclude** |
| --- | --- | --- | --- | --- | --- | --- |
| ZAF_01 | South Africa | Monitoring and evaluation plan for the national strategic plan on HIV, TB and STI (2017 – 2022) | 2017-2022 | yes | yes | **include** |
| ZAF_02 | South Africa | National Strategic Plan for HIV, TB and STIs: 2023-2028: https://hivpreventioncoalition.unaids.org/sites/default/files/attachments/sanac-nsp-2023-2028-web-version.pdf | 2023-2028 | yes | yes | **include** |
| ZAF_03 | South Africa | [Health strategic plan 2020-2025](https://www.health.gov.za/wp-content/uploads/2020/11/depthealthstrategicplanfinal2020-21to2024-25-1.pdf) | 2020-2025 | No | No | exclude |
| ZAF_04 | South Africa | South africa country operational plan (cop/rop) 2020 | 2020 | No | No | exclude |
| ZAF_05 | South Africa | Draft Integrated Strategy on HIV and AIDS 2012–2016 | 2012–2016 | No | No | exclude |
| ZAF_06 | South Africa | South Africa’s national strategic plan for HIV, TB and STIs 2017-2022 | 2017-2022 | old | old | exclude |
| ZAF_07 | South Africa | Regional strategy for HIV prevention, treatment and care and sexual and reproductive health and rights among key populations | 2017 | No | No | exclude |
| SSD_1_ | South Sudan | Republic of South Sudan Country Operational Plan (COP) 2021 | 2021 | No | No | exclude |
| SSD_2 | South Sudan | Sudan National Strategic Plan and Sectrol Plans on HIV/AIDS | 2004-2009 | No | No | exclude |
| SSD_3 | South Sudan | south-Sudan-Signed-COP22-Approval-Memo | 2022 | No | No | exclude |
| SSD_4 | South Sudan | Revised National HIV and AIDS Strategic Plan 2021 – 2023 | 2021 – 2023 | yes | yes | **include** |

**Table S1. Assessment of documents at country-level (Continued)**

**Continued on next page**

| **Document number** | **Country** | **Document title** | **year of publication** | **Describes surveillance approach (Yes/No)** | **Indicators included**  **{Yes/No}** | **include/exclude** |
| --- | --- | --- | --- | --- | --- | --- |
| TZA_1 | Tanzania | Tanzania Country Operational Plan (COP) 2020 | 2020 | No -old | No | exclude |
| TZA_2 | Tanzania | national multi-sectoral strategic framework on HIV AND AIDS 2021/22 - 2025/26 | 2021/22 - 2025/26 | maybe-mostly M&E | yes | **include** |
| TZA_3 | Tanzania | Health sector HIV AND AIDS strategic plan (HSHSP IV) 2017–2022 | 2017–2022 | maybe-mostly M&E | yes | **include** |
| TZA_4 | Tanzania | Tanzania-COP22-Approval-Memo | 2022 | No | No | exclude |
| TZA_5 | Tanzania | Monitoring and Evaluation Plan , Health Sector HIV and AIDs Strategic Plan 2017–2022 | 2017–2022 | yes | yes | **include** |
| UGA_01_ | Uganda | National HIV AND AIDS strategic plan 2015/2016- 2019/2020 | 2015/2016- 2019/2020 | yes | yes | exclude |
| UGA_02 | Uganda | The monitoring and evaluation plan for the national HIV AND AIDS strategic plan 2020/21-2024/25 | 2020-2025 | yes | yes | **include** |
| UGA_03 | Uganda | [National HIV and AIDS Strategic Plan 2020-2025](https://uac.go.ug/index.php?option=com_content&view=article&id=24:hiv-prevention-1123&catid=8&Itemid=101) | 2020-2025 | yes | yes | **include** |
| ZMB_01_ | Zambia | [2022-2026 National Health Strategic Plan](https://www.moh.gov.zm/wp-content/uploads/2023/02/National-Health-Stratergic-Plan-for-Zambia-2022-to-2026-revised-February-2023-lower-resolution.pdf) | 2022-2026 | maybe-mostly M&E | yes | **include** |
| ZMB_02 | Zambia | [National HIV/AIDS Strategic Framework, 2017-2021](https://www.prepwatch.org/resources/national-hiv-aids-strategic-framework-2017-2021/) | 2017-2021 | No update available | yes | exclude |
| ZMB_03 | Zambia | Zambia Country Operational Plan (COP) 2022 | 2022 | yes | No | **include** |
| ZWE_01 | Zimbabwe | Guidelines-for-HIV-Prevention-Testing-and-Treatment-of-HIV-in-Zimbabwe | 2022 | maybe-mostly M&E | No | exclude |
| ZWE_02 | Zimbabwe | Zimbabwe-national-HIV-stategic-plan_2021-2025 | 2021-2025 | yes | yes | **include** |
| ZWE_03 | Zimbabwe | ZIMBABWE Country Operational Plan | 2022 | yes | No | **include** |

**Table S1. Assessment of documents at country-level (Continued)**

**Table S2. Assessment of documents at global or regional-level**

| **Document number** | **Organization or region** | **Document title** | **year of publication** | **Describes surveillance approach (Yes/No)** | **Indicators included**  **{Yes/No}** | **include/exclude** |
| --- | --- | --- | --- | --- | --- | --- |
| GLB_01 | PEPFAR | Monitoring, Evaluation, and Reporting Indicator Reference Guide | 2022 | yes | yes | **include** |
| GLB_02 | PEPFAR | PEPFAR Country Operating Plan: https://www.state.gov/wp-content/uploads/2023/02/PEPFAR-2023-Country-and-Regional-Operational-Plan.pdf | 2024-2025 | yes | No | **include** |
| GLB_03 | PEPFAR | Addendum to the PEPFAR Country Operating Plan (to be released soon |  | yes | No | **include** |
| GLB_04 | PEPFAR | PEPFAR Monitoring and Evaluation Indicator Reference Guide: https://www.state.gov/wp-content/uploads/2019/10/PEPFAR-MER-Indicator-Reference-Guide-Version-2.4-FY20.pdf | 2019 | yes | yes | Exclude - update available |
| GLB_05 | Global Fund | Global Fund Strategy: https://archive.theglobalfund.org/media/11612/strategy_globalfund2023-2028_narrative_en.pdf | 2023-2028 | yes | No | **include** |
| GLB_06 | Global Fund | Key Performance Indicators (KPI) Handbook for the 2023-2028 Strategy https://www.theglobalfund.org/media/12681/strategy_globalfund2023-2028-kpi_handbook_en.pdf | 2023-2028 | yes | No | **include** |

**Continued on next page**

| **Document number** | **Organization or region** | **Document title** | **year of publication** | **Describes surveillance approach (Yes/No)** | **Indicators included**  **{Yes/No}** | **include/exclude** |
| --- | --- | --- | --- | --- | --- | --- |
| GLB_07 | Global Fund | The Global Fund Strategic Framework for Data Use for Action and Improvement at Country Level: https://www.theglobalfund.org/media/8362/me_datauseforactionandimprovement_framework_en.pdf | 2017-2022 | yes | No | **include** |
| GLB_08 | African Union | Catalytic framework to end AIDS, TB and eliminate malaria in Africa by 2030 | 2016 | yes | No | **include** |
| GLB_09 | UNAIDS | Global AIDS Monitoring Plan 2024: https://www.unaids.org/en/resources/documents/2024/global-aids-monitoring-guidelines | 2024 | yes | yes | **include** |
| GLB_10 | UNAIDS | Monitoring HIV impact using population-based surveys | 2015 | yes | yes | **include** |
| GLB_11 | UNAIDS | Global AIDS Strategy: https://www.unaids.org/en/Global-AIDS-Strategy-2021-2026 | 2021-2026 | yes | No | **include** |
| GLB_12 | UNAIDS | Global AIDS Monitoring Framework: https://www.unaids.org/sites/default/files/media_asset/UNAIDS_GAM_Framework_2022_EN.pdf | 2022-2026 | yes | No | **include** |
| GLB_13 | WHO | Consolidated guidelines on person-centred HIV strategic information: strengthening routine data for impact policy brief on harnessing the strength of routine data for HIV surveillance | 2022 | yes | No | **include** |

**Table S2. Assessment of documents at global or regional-level (Continued)**

**Continued on next page**

**Table S2. Assessment of documents at global or regional-level (Continued)**

| **Document number** | **Organization or region** | **Document title** | **year of publication** | **Describes surveillance approach (Yes/No)** | **Indicators included**  **{Yes/No}** | **include/exclude** |
| --- | --- | --- | --- | --- | --- | --- |
| GLB_15 | Global programme on AIDS/WHO | Unlinked anonymous screening for the public health surveillance of HIV infections: Proposed international guidelines | 1989 | No | No | Exclude |
| GLB_16 | UNAIDS/WHO | Guidelines for second  generation HIV surveillance | 2000 |  |  | Exclude |
| GLB_17 | UNAIDS/WHO | Guidelines for second generation HIV surveillance: an update: Know your epidemic | 2013 | yes | No | **include** |
| GLB_18 | UNAIDS/WHO | Conducting HIV surveillance based on routine programme data among pregnant women attending antenatal clinics | 2015 | yes | No | **include** |
| GLB_19 | WHO | Surveillance of HIV drug resistance in children newly diagnosed with HIV by early infant diagnosis | 2017 | yes | No | **include** |
| GLB_20 | WHO | Consolidated guidelines on personcentred HIV patient monitoring and case surveillance | 2017 | yes | No | **include** |
| GLB_21 | WHO/Europe | HIV/AIDS surveillance in Europe 2022 data -2023 | 2023 | yes | No | **include** |
| GLB_22 | WHO | [Consolidated guidelines on person-centred HIV strategic information strengthening routine data for impact](https://www.who.int/teams/global-hiv-hepatitis-and-stis-programmes/hiv/strategic-information/hiv-surveillance) | 2022 | yes | Yes | **include** |

**Continued on next page**

**Table S2. Assessment of documents at global or regional-level (Continued)**

| **Document number** | **Organization or region** | **Document title** | **year of publication** | **Describes surveillance approach (Yes/No)** | **Indicators included**  **{Yes/No}** | **include/exclude** |
| --- | --- | --- | --- | --- | --- | --- |
| GLB_24 | FIND/WHO | Find & who working group on HIV Incidence assays meeting report | 2016 | No | No | Exclude |
| GLB_25 | Global/regional | Guide to conducting programme reviews for HIV, viral hepatitis and sexually transmitted infections | 2023 | No | No | Exclude |
| GLB_26 | World Bank | [Public Health Surveillance Toolkit](https://documents1.worldbank.org/curated/en/383611468154452352/pdf/563720WP0REPLA00Box349502B00PUBLIC0.pdf) | 2002 |  |  | Exclude |
| GLB_27 | WHO | Framework for an integrated multisectoral response to TB, HIV, STIs and Hepatitis in the WHO Africa Region, 2021-2030 | 2021-2030 | No | No | Exclude |
| GLB_29 | Global Fund | Global Fund M&E Framework: https://www.theglobalfund.org/en/monitoring-evaluation/strategic-monitoring/ | 2023-2028 | N/a | n/a | Exclude |
| GLB_30 | Global Fund | Key Performance Indicators (KPIs)  Handbook for the 2023-2028 Strategy | 2023-2028 | yes | yes | **include** |
| GLB_31 | Global Fund | The Global Fund Strategic  Framework for Data Use for Action  and Improvement at Country Level | 2017-2022 | yes | yes | **include** |
| GLB_32 | Global Fund | Global Fund Monitoring & Evaluation (M&E) Framework including KPIs and the Multi-Year Evaluation Calendar 48 th Board Meeting: https://archive.theglobalfund.org/media/12503/archive_bm48-04-me-framework-kpis-multi-year-evaluation-calendar_report_en.pdf |  | yes | yes | **include** |
| GLB_33 | UNAIDS | [Core indicators for national HIV programmes](https://www.unaids.org/sites/default/files/sub_landing/files/JC1768-Additional_Indicators_v2_En.pdf) | 2007 | old | old | exclude |

**Continued on next page**

**Table S2. Assessment of documents at global or regional-level (Continued)**

| **Document number** | **Organization or region** | **Document title** | **year of publication** | **Describes surveillance approach (Yes/No)** | **Indicators including**  **{Yes/No}** | **include/exclude** |
| --- | --- | --- | --- | --- | --- | --- |
| GLB_34 | WHO | National AIDS programmes, a guide to monitoring and evaluating HIV/AIDS care and support | 2004 | old | old | exclude |
| GLB_35 | WHO | Consolidated guidelines on person-centred HIV strategic information: strengthening routine data for impact policy brief on digital health data | 2022 | No | No | exclude |
| GLB_36 | WHO | Consolidated guidelines on person-centred HIV strategic information: strengthening routine data for impact policy brief on person-centred monitoring to focus prevention and guide programmes | 2022 | No | No | exclude |
| GLB_37 | WHO | Consolidated guidelines on person-centred HIV strategic information: strengthening routine data for impact policy brief on enhancing monitoring of HIV testing and treatment | 2022 | No | No | exclude |
| GLB_38 | WHO | Consolidated guidelines on person-centred HIV strategic information: strengthening routine data for impact policy brief on integrating and strengthening monitoring of differentiated art service delivery | 2022 | No | No | exclude |

**Continued on next page**

| **Document number** | **Organization or region** | **Document title** | **year of publication** | **Describes surveillance approach (Yes/No)** | **Indicators included**  **{Yes/No}** | **include/exclude** |
| --- | --- | --- | --- | --- | --- | --- |
| GLB_39 | WHO | Consolidated guidelines on person-centred HIV strategic information: strengthening routine data for impact policy brief on integrating related infections into HIV surveillance systems | 2022 | No | No | exclude |
| GLB_40 | WHO | surveillance of antiretroviral toxicity, global HIV, hepatitis and stis programme what’s new in person-centred HIV patient and antiretroviral drug toxicity monitoring | 2020 | No | No | exclude |
| GLB_41 | WHO | HIV drug resistance surveillance in countries scaling up pre-exposure prophylaxis | 2020 | No | No | exclude |
| GLB_42 | WHO | Digital adaptation kit for HIV operational requirements for implementing who recommendations in digital systems | 2022 | No | No | exclude |
| GLB_43 | WHO | HIV/AIDS: strategy for the WHO African region | 2013 | No | No | exclude |

**Table S2. Assessment of documents at global or regional-level (Continued)**

|  | **Zimbabwe** | | ***Kenya*** | | ***Lesotho*** | | ***Malawi*** | | ***Ghana*** | |
| --- | --- | --- | --- | --- | --- | --- | --- | --- | --- | --- |
| **Domain** | **Strategic Plan, 202/25** | ***PEPFAR COP22*** | ***Strategic Plan 2021/27*** | ***PEPFAR COP22*** | ***Strategic Plan 2023 - 2028*** | ***PEPFAR COP22*** | ***Strategic Plan 2020/25*** | ***PEPFAR COP22*** | ***Strategic Plan 2021/25*** | ***PEPFAR COP22*** |
| **Current picture of the epidemic** | Zimbabwe seeks to accelerate the HIV response to attain the fast track targets for Ending AIDS as a public health threat by 2025 while sustaining the gains already made. The country has an estimated 1.3 million people living with HIV and had an estimated 17,000 HIV related deaths in 2023. HIV incidence is declining from 13 per 1000 in adults+15 years in 2004 before wide ART scale-up to 1.32 per 1000 adults+15 years in 2023. | Zimbabwe has 1.34 million people living with HIV (PLHIV) ... Annual all-cause deaths among PLHIV have declined over the past decade ... Total annual new HIV infections declined nationally ... Overall HIV prevalence for adults aged 15-49 was 11.8% in 2020, down from 18.1% in 2005. | There were 1.4 million people living with HIV in Kenya in 2023 and an estimated 21,000 AIDS related deaths. HIV incidence among people+15 years old was 4 per1000 in 2004 and is <0.5 per 1000 in 2023. | N/A | Broad aim is to use data science, research and innovation to inform HIV, TB and STI programming, and ensure evidence-based and adaptive responses. broadly says "Utilize modern data science tools to analyze and interpret HIV, TB and STI trends and patterns." without any specific detail | Lesotho is one of the few PEPFAR-supported countries that have achieved the UNAIDS 90-90-90 targets for 2020. PEPFAR Lesotho will continue to support building a framework for programmatic improvement using recent infections along with surveys and programmatic data at every opportunity in COP23 | HIV surveillance is identified as important in tracking gains in HIV control. Following surveys are conducted to check progress: MDHS, MICS and the biological and behavioural biological surveys (BBSS) which are conducted periodically by the National Statistical Office. The MoHP conducts the MPHIA. | Malawi has almost achieved the 95-95-95 goals (88%-98%-97%). Aim to strengthen recency testing to guide targeted response | HIV prevalence is 1.68% and there is a steady decrease in new HIV infections projected to reach just over 9,000 by 2025 |  |
| **What data sources country heavily relies on currently?** | The country relies heavily on facility-based reports, routine health information systems, and program-specific surveys. | N/A | N/A | Routinely collected data EMR | routine mainly through DHIS2 and PHIA | Data from the LePHIA 2020 and UNAIDS Spectrum model form the basis for national planning and estimates..." (Page 8) | Major sources include antenatal clinics (ANC), population-based surveys (e.g., MPHIA), and facility-based data | Routinely collected data and outputs from mathematical models | routine health facility data, population-based surveys;. Other sources include surveillance data (e.g. behavioral and biological surveys) and mortality records and reports |  |

## **Table S3. Narrative synthesis of focus countries**

**Continued on next page**

**Table S3. Narrative synthesis of focus countries (Continued)**

**Continued on next page**

|  | **Zimbabwe** | | ***Kenya*** | | ***Lesotho*** | | Malawi | | ***Ghana*** | |
| --- | --- | --- | --- | --- | --- | --- | --- | --- | --- | --- |
| **Domain** | **Strategic Plan, 202/25** | ***PEPFAR COP22*** | ***Strategic Plan 2021/27*** | ***PEPFAR COP22*** | ***strategic plan 2023 - 2028*** | ***PEPFAR COP22*** | ***strategic plan 2020/25*** | ***PEPFAR COP22*** | ***Strategic plan 2021/25*** | ***PEPFAR COP22*** |
| **What are the identified data (source) gaps (if any)?** | "Need to identify populations at risk in each district by undertaking granular vulnerability analysis to identify the populations contributing to new infections for effective targeting" (p.14) "There is a need for a deep dive into the factors contributing to AIDS deaths and to develop approaches to reduce AIDS deaths" (p.15) ""Lack of data on size estimation and HIV burden among some KPs (p.30) "There is no national M&E system monitoring the key pops" (p30)  Routine HIV programme monitoring tools are paper based and difficult to use | data from private sector, health insurance companies, households, and mission facilities were not captured. | N/A | N/A | Develop an interoperable HIV case-based surveillance system by integrating a unique identifier system for the current eRegister,and link it with the national civil registration and vital statistics to a central national data warehouse. | Other improvements that will continue to be addressed for all sites and ensuring complete entry and reporting will focus on the HTS, registration and pharmacy modules of the eRegister. In addition, COP2022 will focus on the improvement of interoperability of eRegister, DHIS2 with other MOH information systems including the laboratory, pharmacy, and logistics information systems. | N/A | n/a | Special studies should be considered when routine data collection and analysis is inappropriate or not feasible. Data from organizations providing community-based HIV services are also essential. lack of a comprehensive, real-time data system, and challenges in routine monitoring of key populations. |  |

|  | **Zimbabwe** | | ***Kenya*** | | ***Lesotho*** | | Malawi | | ***Ghana*** | |
| --- | --- | --- | --- | --- | --- | --- | --- | --- | --- | --- |
| **Domain** | **Strategic Plan, 202/25** | ***PEPFAR COP22*** | ***Strategic Plan 2021/27*** | ***PEPFAR COP22*** | ***strategic plan 2023 - 2028*** | ***PEPFAR COP22*** | ***strategic plan 2020/25*** | ***PEPFAR COP22*** | ***Strategic plan 2021/25*** | ***PEPFAR COP22*** |
| **Impression of general future direction** | "Shifting from a national to a local response micro-targeting districts and populations (and sub-populations) based on evidence. An analysis of the HIV burden (prevalence, new infections and AIDS-related deaths) and programmes coverage by district and populations will inform a targting strategy and level of human, technical and financial resources investment required for each district... whereas national data is critical in providing a picture of the country's response, in the next five years, the tracking of progress will be district- and population-specific"(p.19) | Zimbabwe will focus on increasing efforts to establish case-based surveillance (CBS) and strengthen data use. Detecting recent HIV infections among all newly diagnosed individuals in near-real-time and establishing a surveillance system to longitudinally track HIV cases has been designated a high priority activity. Need for early detection of potential hot spots to inform subsequent mitigation efforts to reduce HIV incidence | "Kenya aims to end AIDS as a public health threat by 2030 by addressing critical issues affecting the HIV response." The NACC plans to "expand granular data approaches and enhance digital systems." | Shifts in program focus towards a more proactive approach to identify the networks within populations of people living with HIV who have, as yet, not been identified, as well as ensuring continuity of treatment and viral suppression among all that are on antiretroviral therapy (ART), in order to reduce the HIV transmission, morbidity and mortality. | Continue current drive towards expanding access to HIV testing, treatment and preventive services but also prioritisation of interventions to hotspots | Stakeholders were supportive of focusing on the 95-95-95 and 10-10-10 goals and beginning the plans for transitioning to a sustainable model. | No specific mention of future surveillance plan, but aims to imrpove data systems: "multisectoral collaboration and integration of services to enhance data quality and decision-making" | No specific mention of future surveillance plan, | No specific mention of future surveillance plan | N/A |

**Table S3. Narrative synthesis of focus countries (Continued)**

**Continued on next page**

|  | **Zimbabwe** | | ***Kenya*** | | ***Lesotho*** | | Malawi | | ***Ghana*** | |
| --- | --- | --- | --- | --- | --- | --- | --- | --- | --- | --- |
| **Domain** | **Strategic Plan, 202/25** | ***PEPFAR COP22*** | ***Strategic Plan 2021/27*** | ***PEPFAR COP22*** | ***strategic plan 2023 - 2028*** | ***PEPFAR COP22*** | ***strategic plan 2020/25*** | ***PEPFAR COP22*** | ***Strategic plan 2021/25*** | ***PEPFAR COP22*** |
| **What is current and future status of (national) population based surveys** | National surveys were conducted in 2015/16 and in 2020 . Surveys and surveillance: The country lacks an inventory of surveys carried out and those planned to be conducted. Also, there is no clear mobilisation strategy for resource mobilisation for surveys. There is need to implemen regular Bio-Behavioural and Size estimates for Key populations | N/A | Population-based HIV Impact Assessment conducted in 2018 and one currently being planned for start in 2025 | Population-based HIV Impact Assessment conducted in 2018 and one currently being planned for start in 2025 | The *Lesotho Population*-based *HIV* Impact Assessment (LePHIA 2016/17 and another one in 2020) | The *Lesotho Population*-based *HIV* Impact Assessment (LePHIA 2016/17 and another one in 2020) | Two PHIAs done in 2015/16 and in 2020/21. There are concerns around sustainability of these surveys in Malawi "This will need to form part of evidence-based programming if sustained." | not mentioned on future plans | The plan emphasizes using population-based surveys such as the Demographic and Health Survey (DHS) and Multi Indicator Cluster Survey (MICS) to monitor HIV prevalence and evaluate the effectiveness of interventions. | N/A |
| **What new methods or technology used for HIV surveillance:** **HIV recency testing (Comment such as when testing started and coverage in regions)** | N/A | Recency testing is conducted in 1,314 sites reaching 65% coverage. Upon analysis of the recency data that the program has targeted HIV testing outreach services to hotspots | Not specified | Recency testing is expanded to all 40 PEPFAR-supported counties. Expansion of recency will help identify hotspots of new infections to guide a public health response. | Map recent infection hotspots using recency testing data to prioritize a comprehensive package of services, including HTS, PrEP, condoms, ART initiation, and other services | Recency implementation is in all ten districts covering 180 facilities March 2023. All persons newly diagnosed with HIV, age 15 or older, are offered recency testing upon consent. Recency data will be utilized to execute cluster mapping and identify hotspots and also guide prevention programming. | Recency testing mentioned but no details | recent infection surveillance to identify and respond to clusters of new infections with targeted HIV prevention and treatment efforts.... The recency surveillance system was expanded to 253 health facilities in 27 districts to cover 80% of newly diagnosed people living with HIV nationally. | identified as important but not clear if this is currently implemented in Ghana | N/A |

**Table S3. Narrative synthesis of focus countries (Continued)**

**Continued on next page**

|  | **Zimbabwe** | | ***Kenya*** | | ***Lesotho*** | | Malawi | | ***Ghana*** | |
| --- | --- | --- | --- | --- | --- | --- | --- | --- | --- | --- |
| **Domain** | **Strategic Plan, 202/25** | ***PEPFAR COP22*** | ***Strategic Plan 2021/27*** | ***PEPFAR COP22*** | ***strategic plan 2023 - 2028*** | ***PEPFAR COP22*** | ***strategic plan 2020/25*** | ***PEPFAR COP22*** | ***Strategic plan 2021/25*** | ***PEPFAR COP22*** |
| **What new methods or technology used for HIV surveillance:** **Case-based surveillance** | Lack of unique identifiers resulting in weak tracking and reporting of false defaulters. Lack of inter-operability between different electronic strategic information platforms. Planned case-based MTCT tracking mechanisms (pregnant women & new infant infections). Plans to develop and roll-out unique identifier code ( UIC) | CBS linked to recency testing and providing long term follow-up to maximize continuity of treatment and viral load suppression such that re-ignition of the epidemic does not occur. | Not specified | case-based surveillance is operational | Develop an interoperable HIV case-based surveillance system by integrating a unique identifier system for the current eRegister,and link it with the national civil registration and vital statistics to a central national data warehouse. | integration of a unique identifier system for the current eRegister, linking it with national civil registration and vital statistics and the development of a central data repository. This will allow the successful development of a case-based surveillance system for Lesotho and its linkage with mortality surveillance and drug resistance surveillance. | Case-based surveillance is being implemented: "The NSP 2020-2025 includes plans to strengthen case-based surveillance using EMR and facility-based reporting systems" | Based on the EMR system. | Innovations include the adoption of the Ghana Key Population Unique Identification System (GKPUIS) for tracking services provided to key populations and the expanded use of differentiated HIV Testing Services (dHTS) | N/A |
| **Subnational modelling (esp. new infections/VL)** | National-level estimates of new infections uses Spectrum. Subnational estimate available estimated per province and district. Subnational estimates made from The Incidence Patterns Modelling (NAC and UNAIDS, 2017) and NAOMI model. | PEPFAR used 2021 subnational HIV estimates from the UNAIDS NAOMI model and host country treatment program data to recalibrate the national HIV epidemic. | N/A | Models such as Spectrum and Naomi are used for subnational estimates and disaggregations by age | N/A | N/A | Naomi model used for subnational etsimation. Estimates of subnational new HIV infections presented in national map | Spectrum/Naomi estimates used for 2022 projections | Spectrum used for national estimates of new infections with disaggregation by age. No subnationalprojections shown | N/A |

**Table S3. Narrative synthesis of focus countries (Continued)**

**Continued on next page**

|  | **Zimbabwe** | | ***Kenya*** | | ***Lesotho*** | | Malawi | | ***Ghana*** | |
| --- | --- | --- | --- | --- | --- | --- | --- | --- | --- | --- |
| **Domain** | **Strategic Plan, 202/25** | ***PEPFAR COP22*** | ***Strategic Plan 2021/27*** | ***PEPFAR COP22*** | ***strategic plan 2023 - 2028*** | ***PEPFAR COP22*** | ***strategic plan 2020/25*** | ***PEPFAR COP22*** | ***Strategic plan 2021/25*** | ***PEPFAR COP22*** |
| **Disaggregated modelling/reporting (esp. new infections/VL)** | Disaggregations of new HIV infections available by age and key populations such as PWID, MSM, FSW, as well as by marital status and circumcision status | N/A |  |  | Currently uses UNAIDS specturm model to show diaggregations in new HIV infections by gender and age (0-14, 15-24, 15+). | VL suppression rates howed by by gender and age (0-14, 15-24, 25+). Also in military populations. VL disaagregations also by district | N/A | the PEPFAR Malawi team will conduct a KP size estimation study to inform scale and design of future KP programs | Spectrum used for national estimates of new infections with disaggregation by age. No subnational projections shown | N/A |
| **Mortality measurement** | Model estimates of mortality available disaggregated by district and age. | N/A | Mortality data is derived from "Kenya HIV Estimates" and programmatic monitoring | Mortality rates are tracked as part of the 95-95-95 targets (diagnosis, treatment, and viral suppression goals) | documents not reporting on mortality measurement | N/A | N/A | N/A | N/A | N/A |
| **Genomic surveillance (Drug resistance or transmission networks)** | Genomic surveillance, including studies on drug resistance and STI transmission networks, is still underdeveloped. | N/A | Monitoring resistance is identified as important | Not explicitly mentioned | Only drug resistance monitoring done during PHIA | HIVDR surveillance activities will focus on patients failing on TLD. HIV Drug Resistance surveillance may include (i)use of remnant samples routinely collected for patient care, (ii) Use of Cyclical Acquired Drug Resistance (CADRE) by obtaining samples and minimum epi data from Lesotho’s viral load testing platforms. | The monitoring of HIV drug resistant strains will be conducted regularly | No direct mention of genomic surveillance for drug resistance or transmission networks was found in the extract. Laboratory strengthening is mentioned, but it does not specify genomic methods. | Drug resistance monitoring through genotyping is part of routine surveillance. However, there is no specific mention of genomic studies on transmission networks | N/A |

**Table S3. Narrative synthesis of focus countries (Continued)**

**Continued on next page**

|  | **Zimbabwe** | | ***Kenya*** | | ***Lesotho*** | | Malawi | | ***Ghana*** | |
| --- | --- | --- | --- | --- | --- | --- | --- | --- | --- | --- |
| **Domain** | **Strategic Plan, 202/25** | ***PEPFAR COP22*** | ***Strategic Plan 2021/27*** | ***PEPFAR COP22*** | ***strategic plan 2023 - 2028*** | ***PEPFAR COP22*** | ***strategic plan 2020/25*** | ***PEPFAR COP22*** | ***Strategic plan 2021/25*** | ***PEPFAR COP22*** |
| **Other** |  |  |  |  |  |  |  |  |  |  |
| **Observations about viral load testing capacity and performance** | Viral load coverage among adults  increased from 6% in 2015 to 44% in 2018 but still "Long turnaround time for viral load test results and low viral load testing coverage" (p52) | N/A | N/A | N/A | Viral load coverage (VLC) has increased from 61% in 2018 to 91% in 2022. Challenges such as long turn around times mentioned | N/A | In June 2019, viral load coverage was 78% in adults and 70% in children. Some challenges that need to be addressed such as delays in TAT for VL test results and high workloads at molecular laboratories | N/A | Reducing turnaround time for viral load tests by enhancing  the laboratory information management system (LIMS). Viral load testing cost was $138,282,410 of total $661,562,182: viral load testing alone estimated to take 20% of all planned expenditure on HIV programming | N/A |
| **Other innovations** | HIV incidence study in prisoners Service delivery integration with HIV services - STI, TB, NCDs. Aim to pilot innovative big-data and artificial intelligence-driven real-time monitoring of the HIV epidemic and/or response. | N/A | N/A | Machine learning applications are used to identify patient characteristics linked to higher likelihood of early treatment interruption. | N/A | N/A | N/A | N/A | N/A | N/A |

**Table S3. Narrative synthesis of focus countries (Continued)**

## **Table S4 Detailed country extraction: Ghana**

| **Ghana** |  |  |  |  |
| --- | --- | --- | --- | --- |
|  | **Mentioned in strategy/surveillance** | **Implemented** | **Frequency** | **Data sources** |
| **Risk** |  |  |  |  |
| Household surveys on behaviour or stigma (e.g DHS, MICS) | Yes  Ghana DHS and MICS stated as source of various HIV indicators (*Ghana AIDS Commission. (National HIV & AIDS Strategic Plan 2021-2025), page 23*) | Fully implemented | Conducted every 5 to 6 years since 1988, latest survey in 2022  ICF International. Ghana: Demographic and Health Surveys (DHS) [Internet]. The DHS Program; [cited 2025 Mar 10]. Available from: <https://dhsprogram.com/Countries/Country-Main.cfm?ctry_id=14> | Survey |
| Behavioral surveys (inc.key pops) | Yes  Ghana Integrated Biological and Behavioral Surveillance Surveys (IBBSS) stated as source of various HIV indicators (*Ghana AIDS Commission. (National HIV & AIDS Strategic Plan 2021-2025), page 26*) | Fully implemented | IBBSS in Ghana are conducted periodically among key populations such as female sex workers (FSWs) and men who have sex with men (MSM). The frequency of these surveys has varied over time. For **FSWs, IBBSS have been conducted every two to three** years in cities like Accra and Kumasi. For MSM, the first national IBBSS, known as the Ghana Men's Study I (GMS I), was conducted in 2011, followed by the Ghana Men's Study II (GMS II) in 2017 ( sample size of 4,095 MSM). A third round for MSM was conducted in 2022/23. | Surveys |
| STI surveillance | Yes  Uses routine data to estimate “Percentage of patients receiving diagnosis and treatment for STIs according to national guidelines” (including advice on prevention) (Ghana AIDS Commission. (National HIV & AIDS Strategic Plan 2021-2025), page 93) | Fully implemented | Ongoing | Routine data |
| **HIV infection** |  |  |  |  |
| HIV incidence (modelling) | No  Only estimates of new infections presumably from Spectrum presented (*Ghana AIDS Commission. (National HIV & AIDS Strategic Plan 2021-2025), page 19*) | Fully implemented | Annually | Mathematical modelling |
| Recency surveys (general [PHIA]and subgroups e.g. AGYW) | No  Not mentioned | Not documented |  |  |
| Early infant diagnosis | Yes  Programme tracks EID Coverage in HIV Exposed Infants (HEI)  (National HIV & AIDS Strategic Plan 2021-2025), page 38) | Fully implemented | Ongoing | Routine data |
| Cohort studies for HIV incidence | No | Not documented | Not documented | Research |
| **HIV morbidity** |  |  |  |  |
| ANC surveillance (+Sentinel), PMTCT | Yes  Use of ANC registers and ANC prevalence estimate stated.  National HIV & AIDS Strategic Plan 2021-2025), page 10) | Fully implemented | Ongoing | Routine data |
| Population based impact surveys | Not mentioned | Not documented | Not documented |  |
| Case-Based Surveillance (+advanced HIV, LIMS/NHLS data) | Not mentioned  “Reducing turnaround time for viral load tests by enhancing the laboratory information management system (LIMS) to enable remote log-in of the viral load and EID results and downloading of same at facility levels as well as the full implementation of the LIMS-EMR interface for direct transmission of viral load and EID results from PCR labs to the patient’s electronic medical records” | Not documented |  |  |
| Key population surveys (size and prevalence) | Yes  Estimated sizes of key population and estimates of HIV prevalence stated.  National HIV & AIDS Strategic Plan 2021-2025), page 10) | Fully implemented | Estimated from IBBSS, see above | Survey |
| HIV ART Care records (Number on ART, ART outcomes) | Yes  Number of people on ART presented  National HIV & AIDS Strategic Plan 2021-2025), page 52) | Fully implemented | Ongoing | Routine data |
| HIV drug resistance surveys | Yes  *“As part of the monitoring process, the country will conduct ARV drug resistance and toxicity studies.”*  National HIV & AIDS Strategic Plan 2021-2025), page 55) | Fully implemented | First drug resistance survey conducted between April 2016 and June 2017.  Sylverken, A. A., Kwarteng, A., Twumasi-Ankrah, S., Owusu, M., Arthur, R. A., Dumevi, R. M., ... & Yeboah-Manu, D. (2021). The burden of drug resistance tuberculosis in Ghana; results of the First National Survey. PLOS ONE, 16(6), e0252819 | Survey |
| Pharmacovigilance (e.g. birth defects surveillance) | Yes  *“As part of the monitoring process, the country will conduct ARV drug resistance and toxicity studies.”*  National HIV & AIDS Strategic Plan 2021-2025), page 55) | Not documented | Not documented | Survey |
| HIV-associated infections (e.g Cervical cancer, TB, Hepatitis) | Yes  *“The country will continue to prioritize TB/HIV activities to combat the dual infection*  *of HIV and TB. The core activities will include TB screening for all PLHIV; timely TB diagnosis; early initiation and treatment completion for PLHIV; scale up of TPT (TB*  *Preventive Treatment); transition to more optimal regimens such as 3HP (once-weekly isoniazid-rifampicin for 12 weeks); sustained joint TB/HIV programming and monitoring including scaling up One Stop Shop (OSS) for PLHIV dually infected by TB and HIV.”*  National HIV & AIDS Strategic Plan 2021-2025), page 55) | Partially implemented | Not stated | Routine data |
| HIV prevalence in blood donors | Yes  As part of blood safety procedures.  National HIV & AIDS Strategic Plan 2021-2025), page 97) | Fully implemented | Not stated | Routine data |
| **Mortality** |  |  |  |  |
| Mathematical modelling | No  AIDS deaths reported, presumably form modelling.  National HIV & AIDS Strategic Plan 2021-2025), page 3) | Fully implemented | Not stated | Modelling |
| Vital registration (national, DHSS) | No | Not documented | Not documented |  |
| Community mortality reporting systems/surveys | No | Not documented | Not documented |  |
| Facility-based mortality reporting systems | No | Not documented | Not documented |  |

Abbreviations: DHS=Demographic and Health survey, MICS=Multiple Indicator Survey, STI=Sexually transmitted infections, AGYW=Adolescent Girls and Young Women, ANC=Antenatal Clinic, PMTCT=Prevention of mother to child transmission, LIMS=Laboratory information management system, NHLS=National health laboratory System, DHSS=Demographic health surveillance site

## **Table S5 Detailed country extraction: Kenya**

| **Kenya** |  |  |  |  |
| --- | --- | --- | --- | --- |
| **Risk** | **Mentioned in strategy/surveillance** | **Implemented** | **Frequency** | **Data sources** |
| Household surveys on behaviour or stigma (e.g DHS, MICS) | Yes  Stated in national strategy  “*Kenya has implemented periodic*  *national population-based surveys such as the Kenya Demographic and Health Survey (KDHS), Kenya AIDS*  *Indicator Survey (KAIS), Integrated Biological and Behavioural Studies (IBBS), Size Estimates for at risk groups, HIV Stigma and Discrimination Survey, Kenya Population-Based HIV Impact Assessment (KENPHIA)”* | Fully implemented | Not stated in national strategy but conducted 5 yearly, recently done in 2022 | Survey |
| Behavioral surveys (inc.key pops) | Yes  Mentioned in national strategy | Fully implemented | Not stated in national strategy, but conducted in 2010/11 and one recent in 2024 targeting 10,000 participants across four key populations (Female Sex Workers, Men who have Sex with Men, People Who Inject Drugs, and Transgender individuals) from nine counties | Survey |
| STI surveillance | Yes  Stated in national strategy, to *institutionalise periodic surveillance for STI and Viral hepatitis including embedment into special and population level impact surveys* | Fully implemented | Not stated | Routine programme data and surveys suggested |
| **HIV infection** |  |  |  |  |
| HIV incidence (modelling) | Yes  HIV incidence estimation is mainly from mathematical models | Fully implemented | Annually | Mathematical models |
| Recency surveys (general and subgroups e.g. AGYW) | Yes  But national strategy document does not mention this, COP documents mention this | Fully implemented | Not stated | Not stated |
| Early infant diagnosis | Yes  National strategy states  “HIV testing infrastructure (HTS, Recency testing, EID,  VL and DR) has to be scaled up and expanded”. COP 22 document stated that uptake of EID is suboptimal, negatively impacting provision of HIV prevention and treatment services among HIV exposed infants. In FY22Q2 based on EID data for that quarter, 362 infants (3.6%) tested HIV positive by 12 months, bearing in mind that coverage was less than 50%. There is a remaining backlog of EID tests sampled previously when viral load reagents were unavailable. | Fully implemented | Not stated | Routine programme data |
| Cohort studies for incidence | Yes  HIV incidence estimation is mainly from mathematical models, national strategy does not mention cohort studies. COP document supports cohort studies “Support RV 393 study a prospective, observational cohort study of 600 HIV uninfected adult men and women at risk of HIV infection in Kisumu County, Kenya” and Support AFRICOS HIV Cohort and Pediatric Viral Load Studies and subnational population cohort studies conducted via health and demographic surveillance systems | Partially implemented | Not stated | Research studies |
| **HIV morbidity** |  |  |  |  |
| ANC surveillance (+Sentinel), PMTCT | Yes  To identify new infections among pregnant women, all pregnant women attending ANC will be tested during their first ANC visit and subsequently during pregnancy and postnatal period as per the national MOH guidelines. Sustain leadership, advocacy and coordination of the eMTCT programme | Fully implemented | Not stated | Routine programme data |
| Population based impact surveys | Yes  Conduct periodic population-based surveys for HIV, STIs and other comorbidities. | Fully implemented | Not stated | PHIAs (Population-based HIV Impact Assessment) conducted in 2018 and one currently being planned for start in 2025 |
| Case-Based Surveillance (+advanced HIV, LIMS/NHLS data) | Yes  Strengthen surveillance for HIV, STIs and other comorbidities including  case-based, recency, STIs, drug resistance, mortality, phylogenetic, size estimates, injection, and commodities among others. | Fully implemented | Not stated | Routine programme data |
| Key population surveys (size and prevalence) | Yes  A separate policy for the prevention of HIV infections among key populations in Kenya stated mapping of key populations done by NASCOP (2012) revealed that these numbers were too high to ignore. The mapping estimates more than 133000 FSWs, 13000 MSM and 18000 PWID in Kenya. Methods of estimation not stated. | Fully implemented | Not stated | Data sources not stated |
| HIV ART Care records (Number on ART, ART outcomes) | Yes | Fully implemented | Not stated | Routine |
| HIV drug resistance surveys | Yes | Yes national survey conducted in 2018 | Not stated | Survey |
| Pharmacovigilance (e.g. birth defects surveillance) | No  National strategy does not mention, but Subnational studies under Academic Model Providing Access to Healthcare (AMPATH) in western Kenya | Partially implemented | Not stated | Survey |
| HIV-associated infections (e.g Cervical cancer, TB, Hepatitis) | Yes  Strengthen screening and management of TB, cervical cancers and other NCD’s and comorbidities among PLHIV.  Strengthen estimation and modelling for HIV, STIs and other comorbidities. Analysis on some of the diseases (TB) is done. | Partially implemented | Not stated | Routine data sources |
| HIV prevalence in blood donors | Yes  Strengthen the capacity of the national blood safety programme to provide safe blood. HIV estimated from blood donor services not used. | Fully implemented | Not stated | Routine data sources |
| **Mortality** |  |  |  |  |
| Mathematical modelling | Yes | Fully implemented | Annually | Mathematical models |
| Vital registration (national, DHSS) | No  Other documents show existence of vital registration system and use to estimate HIV mortality | Partially implemented | Not stated | Surveys |
| Community mortality reporting systems/surveys | No  Not mentioned in national strategy but evidence of subnational Mortality surveillance conducted within health and demographic surveillance system (HDSS) sites | Partially implemented | Not stated | Surveys |
| Facility-based mortality reporting systems | No  National HIV strategy does not mention but HIV mortuary surveillance study was conducted in Nairobi and Kisumu, for ascertainment of HIV status. | Partially implemented | Not stated | Surveys |

Abbreviations: DHS=Demographic and Health survey, MICS=Multiple Indicator Survey, STI=Sexually transmitted infections, AGYW=Adolescent Girls and Young Women, ANC=Antenatal Clinic, PMTCT=Prevention of mother to child transmission, LIMS=Laboratory information management system, NHLS=National health laboratory System, DHSS=Demographic health surveillance site

## **Table S6 Detailed country extraction: Lesotho**

| **Lesotho** |  |  |  |  |
| --- | --- | --- | --- | --- |
|  | **Mentioned in strategy/surveillance** | **Implemented** | **Frequency** | **Data sources** |
| **Risk** |  |  |  |  |
| Household surveys on behaviour or stigma (e.g DHS, MICS) | Yes  LePHIA and DHS stated as sources of indicators.  *National AIDS Commission of Lesotho. Lesotho National HIV and AIDS Strategic Plan 2023–2028. Lesotho: National AIDS Commission; 2023. p. 115.* | Fully implemented | Lesotho Population-based HIV Impact Assessment (LePHIA) was conducted in 2016/17 and 2020  Most recent Demographic and Health Survey (DHS) conducted in Lesotho is the 2023-24 | Survey |
| Behavioral surveys (inc.key pops) | Yes  IBBS stated as sources of indicators  *National AIDS Commission of Lesotho. Lesotho National HIV and AIDS Strategic Plan 2023–2028. Lesotho: National AIDS Commission; 2023. p. 114.* | Fully implemented | Last conducted in 2019 | Survey |
| STI surveillance | Yes  Utilize modern data science tools to analyze and interpret HIV, TB and STI trends and patterns.  *National AIDS Commission of Lesotho. Lesotho National HIV and AIDS Strategic Plan 2023–2028. Lesotho: National AIDS Commission; 2023. p. 102.* | Fully implemented | Ongoing | Routine programme data |
| **HIV infection** |  |  |  |  |
| HIV incidence (modelling) | *Yes*  Spectrum outputs of new HIV infection presented.*National AIDS Commission of Lesotho. Lesotho National HIV and AIDS Strategic Plan 2023–2028. Lesotho: National AIDS Commission; 2023. p. 23.* | Fully implemented | Annually, ongoing | Mathematical models |
| Recency surveys (general [PHIA]and subgroups e.g. AGYW) | Yes  Map recent infection hotspots using recency testing data to prioritize a comprehensive package of services, including HTS, PrEP, condoms, ART initiation, and other services  National AIDS Commission of Lesotho. Lesotho National HIV and AIDS Strategic Plan 2023–2028. Lesotho: National AIDS Commission; 2023. p. 69. | Fully implemented | Ongoing | Not stated |
| Early infant diagnosis | Yes  Coverage of EID presented.  National AIDS Commission of Lesotho. Lesotho National HIV and AIDS Strategic Plan 2023–2028. Lesotho: National AIDS Commission; 2023. p. 29. | Fully implemented | Ongoing | Routine programme data |
| Cohort studies for HIV incidence | No | Not documented | Not stated | Not stated |
| **HIV morbidity** |  |  |  |  |
| ANC surveillance (+Sentinel), PMTCT | Yes  ANC testing and PMTCT coverage presented.  *National AIDS Commission of Lesotho. Lesotho National HIV and AIDS Strategic Plan 2023–2028. Lesotho: National AIDS Commission; 2023. p. 29.* | Fully implemented | Ongoing | Routine programme data |
| Population-based impact surveys | Yes  LePHIA and DHS stated as sources of indicators.  *National AIDS Commission of Lesotho. Lesotho National HIV and AIDS Strategic Plan 2023–2028. Lesotho: National AIDS Commission; 2023. p. 115* | Fully implemented | The Lesotho Population-based HIV Impact Assessment (LePHIA 2016/17 and another one in 2020) | Survey |
| Case-Based Surveillance (+advanced HIV, LIMS/NHLS data) | Yes  "Develop an interoperable HIV case-based surveillance system by integrating a unique identifier system for the current eRegister, and link it with the national civil registration and vital statistics to a central national data warehouse."  *National AIDS Commission of Lesotho. Lesotho National HIV and AIDS Strategic Plan 2023–2028. Lesotho: National AIDS Commission; 2023. p. 102* | Fully implemented | Ongoing | Routine programme data |
| Key population surveys (size and prevalence) | Yes  Prevalence in key populations stated.  *National AIDS Commission of Lesotho. Lesotho National HIV and AIDS Strategic Plan 2023–2028. Lesotho: National AIDS Commission; 2023. p. 21* | Fully implemented | Not stated | Data sources not stated |
| HIV ART Care records (Number on ART, ART outcomes) | Yes  ART coverage presented.  *National AIDS Commission of Lesotho. Lesotho National HIV and AIDS Strategic Plan 2023–2028. Lesotho: National AIDS Commission; 2023. p. 31* | Fully implemented | Ongoing | Routine |
| HIV drug resistance surveys | Yes  Conduct drug resistance testing (DRT) for those on integrase strand trans­fer inhibitors (INSTIs) and protease inhibitors (PIs) regimen with confirmed failure  *National AIDS Commission of Lesotho. Lesotho National HIV and AIDS Strategic Plan 2023–2028. Lesotho: National AIDS Commission; 2023. p. 70* | Fully implemented | Drug resistance survey conducted in 2018 | Survey |
| Pharmacovigilance (e.g. birth defects surveillance) | Not stated | Not documented | Not documented | Not stated |
| HIV-associated infections (e.g Cervical cancer, TB, Hepatitis) | Yes  Data science, research, and innovation for precision HIV, TB and STI programming.  Integration of health services will be emphasized, addressing the syndemic of HIV, STIs, cervical cancer, viral hepatitis, substance use, mental health disorders, and non-communicable diseases.  *National AIDS Commission of Lesotho. Lesotho National HIV and AIDS Strategic Plan 2023–2028. Lesotho: National AIDS Commission; 2023. p. 94* | Partially implemented | Ongoing | Routine data |
| HIV prevalence in blood donors | No | Fully implemented | Not stated | Not stated |
| **Mortality** |  |  |  |  |
| Mathematical modelling | Yes  Model outputs of mortality presented.  National AIDS Commission of Lesotho. Lesotho National HIV and AIDS Strategic Plan 2023–2028. Lesotho: National AIDS Commission; 2023. p. 23 | Fully implemented | Annually, ongoing | Mathematical models |
| Vital registration (national, DHSS) | Yes  Develop an interoperable HIV case-based surveillance system by integrating a unique identifier system for the current eRegister,and link it with the national civil registration and vital statistics to a central national data warehouse.  *National AIDS Commission of Lesotho. Lesotho National HIV and AIDS Strategic Plan 2023–2028. Lesotho: National AIDS Commission; 2023. p. 102* | Not documented | Not documented | Not stated |
| Community mortality reporting systems/surveys | No | Not documented | Not documented | Not stated |
| Facility-based mortality reporting systems | No | Not documented | Not documented | Not stated |

Abbreviations: DHS=Demographic and Health survey, MICS=Multiple Indicator Survey, STI=Sexually transmitted infections, AGYW=Adolescent Girls and Young Women, ANC=Antenatal Clinic, PMTCT=Prevention of mother to child transmission, LIMS=Laboratory information management system, NHLS=National health laboratory System, DHSS=Demographic health surveillance site

## **Table S7 Detailed country extraction : Malawi**

| **Malawi** |  |  |  |  |
| --- | --- | --- | --- | --- |
|  | **Mentioned in strategy/surveillance** | **Implemented** | **Frequency** | **Data sources** |
| **Risk** |  |  |  |  |
| Household surveys on behaviour or stigma (e.g DHS, MICS) | Yes  *“MDHS, MICS and the biological and behavioural biological surveys (BBSS) which are conducted periodically by the National Statistical Office. In addition to this, the MoHP conducts the MPHIA which, among other indicators collects data on global targets. This will need to form part of evidence-based programming if sustained”* | Fully implemented | 4-5 years,  Last survey conducted in 2024 | survey |
| Behavioral surveys (inc.key pops) | Yes | Fully implemented | Draft HIV surveillance proposed Every 2 years, last conducted in 2013-14 and 2019-2020 | survey |
| STI surveillance | Yes | Fully implemented | Quarterly | Routine programme data |
| **HIV infection** |  |  |  |  |
| HIV incidence (modelling) | Yes  HIV incidence estimation is mainly from mathematical models | Fully implemented | Annually | Mathematical models |
| Recency surveys (general and subgroups e.g. AGYW) | Yes  Conduct continuous point-of-care recency testing at HTC for newly-tested HIV positive clients (Draft surveillance plan) | Fully implemented | Not stated | Not stated |
| Early infant diagnosis | Yes  Analyse routinely collected early infant diagnosis of HIV (EID) data and include results in Integrated HIV Program reports. COP 22 document states that the program has seen improvement in EID coverage over the last several years and approaching the target of 95%. We also continue to see low early transmission rates below 2% in COP21 Q1. Overall, 96% of newly diagnosed infants were confirmed on ART in COP21 Q1. | Fully implemented | Quarterly | Routine programme data |
| Cohort studies | No | Not documented | Not documented | Not stated |
| **HIV morbidity** |  |  |  |  |
| ANC surveillance (+Sentinel), PMTCT | Yes | Fully implemented | Quarterly | Routine programme data |
| Population based impact surveys | Yes  *MDHS, MICS and the biological and behavioural biological surveys (BBSS) which are conducted periodically by the National Statistical Office. In addition to this, the MoHP conducts the MPHIA which, among other indicators collects data on global targets. This will need to form part of evidence-based programming if sustained* | Fully implemented | 4-5 years | PHIAs (2015–2016) and (2020–2021) |
| Case-Based Surveillance (+advanced HIV, LIMS/NHLS data) | Yes | Fully implemented | Quarterly | Routine programme data |
| Key population surveys (size and prevalence) | Yes  Estimated for Female sex workers (22,152), MSM (3,904) and Transgender people (407). KP implementing organizations: Population size estimate for each KP sub-group. Methods of estimation not stated. | Fully implemented | Not stated | Data sources not stated |
| HIV ART Care records (Number on ART, ART outcomes) | Yes | Fully implemented | Quarterly | Routine |
| HIV drug resistance surveys | Yes  Activity 3.1.1.3.6 Intensify Monitoring of Drug Resistance | Fully implemented | Draft strategy recommends Every 2 years, national HIV drug resistance survey conducted in 2018 | Infant HIVDR Survey: An HIVDR survey was conducted among infants aged 18 months and young in 2016/17  ANC/Pregnant Women HIVDR Survey in 2016  WHO drug resistance survey 2016 |
| Pharmacovigilance (e.g. birth defects surveillance) | Yes  *The MoHP will also support Pharmacovigilance*  *activities that are implemented through the PMRA. This will include improving knowledge in Pharmacovigilance through the continuous training of health staff in the clinical HIV guidelines. There will also be establishment of 10 sentinel sites, 5 of which will be the referral hospitals and the other 5 will be a district hospital in each zone where patients will be more closely followed up.*  *Activity 8.5.1.4.1 Birth defects surveillance* | Fully implemented | Not stated | Surveys |
| HIV-associated infections and comorbidities (e.g Cervical cancer, TB, Hepatitis) | Yes  *Stated as programme activity 4.1.1.3.1 to Expand death audits to all hospital, Analyse TB/HIV co-infection data and include results in quarterly Integrated HIV Program reports (draft surveillance plan)*  *To reduce morbidity and mortality among people living with HIV including other disease related conditions (opportunistic infections, HIV related cancers, NCD, HIV-Hepatitis coinfection, mental health)* | Partially implemented | Quarterly | Routine data |
| HIV prevalence in blood donors | Yes  Stated as Activity *1.9.1.1.2 Provide equipment for blood donor mobilization, blood collection,* *blood testing and blood storage* | Fully implemented | Not stated, presumably on-going | Annually |
| **Mortality** |  |  |  |  |
| Mathematical modelling | Yes  *Outputs of spectrum estimates on mortality shown* | Fully implemented | Annually | Mathematical models |
| Vital registration (national, DHSS) | Yes  Stated as Activity 8.5.1.1.4  *Conduct joint MoHP and NRB national monitoring exercise of Civil registration and vital Statistics activities (both birth and death registration) to all districts* | Not documented | Not documented | Not stated |
| Community mortality reporting systems/surveys | Yes  Stated as programme  *activity 8.5.1.1.21 Roll out health facility based and community based (all districts) death Registration, Scale up the CHIS to Health Surveillance Assistants (HSA) & HIV* | Not documented | Not documented | Not stated |
| Facility-based mortality reporting systems | Yes  *Stated as programme activity 4.1.1.3.1 to Expand death audits to all hospital*  *8.5.1.1.15 Institutionalize the birth and death registration in the MoHP* | Not documented | Not documented | Not stated |

Abbreviations: DHS=Demographic and Health survey, MICS=Multiple Indicator Survey, STI=Sexually transmitted infections, AGYW=Adolescent Girls and Young Women, ANC=Antenatal Clinic, PMTCT=Prevention of mother to child transmission, LIMS=Laboratory information management system, NHLS=National health laboratory System, DHSS=Demographic health surveillance site

## **Table S8 Detailed country extraction : Zimbabwe**

| **Zimbabwe** |  |  |  |  |
| --- | --- | --- | --- | --- |
|  | **Mentioned in strategy/surveillance** | **Implemented** | **Frequency** | **Data sources** |
| **Risk** |  |  |  |  |
| Household surveys on behaviour or stigma (e.g DHS, MICS) | Yes  ZDHS, MICS and ZIMPHIA stated as key sources of indicators  *Ministry of Health and Child Care, Zimbabwe. Zimbabwe National HIV and AIDS Strategic Plan 2021–2025. National AIDS Council; 2020, page 96* | Fully implemented | ZDHS is carried out every 5 years, last delayed and done in  2023–24 ZDHS (previous: 2015) → 8-year gap (likely due to COVID-19 disruptions).  MICS also conducted every 5 years, last conducted in 2019. | Survey |
| Behavioral surveys (inc.key pops) | Yes  Implementing regular Bio-Behavioural and Size estimates for Key populations  *Ministry of Health and Child Care, Zimbabwe. Zimbabwe National HIV and AIDS Strategic Plan 2021–2025. National AIDS Council; 2020, page 81* | Fully implemented | HIV and STI biobehavioral survey  among men who have sex with men, transgender women, and genderqueer individuals in Zimbabwe conducted in 2018–2019  *HIV and STI Biobehavioral Survey among Men Who Have Sex with Men, Transgender Women, and Genderqueer Individuals in Zimbabwe - Final Report. New York: ICAP at Columbia University; 2020.* | Surveys |
| STI surveillance | Yes  STI surveillance is also being implemented through monthly case-based reporting and ANC surveillance  Ministry of Health and Child Care, Zimbabwe. Zimbabwe National HIV and AIDS Strategic Plan 2021–2025. National AIDS Council; 2020, page 54 | Fully implemented | Ongoing | Routine data |
| **HIV infection** |  |  |  |  |
| HIV incidence (modelling) | Yes  Spectrum AND Naomi estimates of new infections presented.  *Zimbabwe National HIV and AIDS Strategic Plan 2021–2025. National AIDS Council; 2020, page 13* | Fully implemented | Ongoing, annual estimates | Mathematical modelling |
| Recency surveys (general [PHIA]and subgroups e.g. AGYW) | Not stated  But COP document outlines: Recency testing is conducted in 1,314 sites reaching 65% coverage. Upon analysis of the recency data that the program has, the program has targeted HIV testing outreach services to hotspots when selecting geographical areas with higher need. Additionally, the recency testing data are used in combination with other data (e.g., PHIAs, historic program performance, UNAIDS estimates, etc.). | Fully implemented | Not stated | NA |
| Early infant diagnosis | Yes  Implemented but challenges states: “Long turn-around time for VL and EID with some sites reporting a TAT of 6 months”  *Zimbabwe National HIV and AIDS Strategic Plan 2021–2025. National AIDS Council; 2020, page 13* | Fully implemented | Ongoing | Routine data |
| Cohort studies for HIV incidence | Not stated  But other local research shows work estimating rates of HIV seroconversion in a cohort of female sex workers DOI: [10.1016/S2352-3018(23)00110-8](https://doi.org/10.1016/s2352-3018(23)00110-8) | Partially implemented | Not stated | NA |
| **HIV morbidity** |  |  |  |  |
| ANC surveillance (+Sentinel), PMTCT | Yes  Establishing case-based MTCT tracking mechanisms at facility and community levels through an electronic system and using community actors to ensure early booking for ANC, tracking of HIV negative pregnant women for HIV retesting and ensuring adherence to ART at ANC and PNC periods  *Zimbabwe National HIV and AIDS Strategic Plan 2021–2025. National AIDS Council; 2020, page 58* | Fully implemented | Ongoing | Routine data |
| Population-based impact surveys | Yes  ZDHS, MICS and ZIMPHIA stated as key sources of indicators  Ministry of Health and Child Care, Zimbabwe. Zimbabwe National HIV and AIDS Strategic Plan 2021–2025. National AIDS Council; 2020, page 96 | Fully implemented | PHIA surveys were conducted in 2015/16 and in 2020  [Zimbabwe - PHIA Project](https://phia.icap.columbia.edu/countries/zimbabwe/) | Surveys |
| Case-Based Surveillance (+advanced HIV, LIMS/NHLS data) | Yes  Establishing case-based MTCT tracking mechanisms at facility and community levels through an electronic system and using community actors to ensure early booking for ANC, tracking of HIV negative pregnant women for HIV retesting and ensuring adherence to ART at ANC and PNC periods.  But also challenges: "Lack of unique identifiers resulting in weak tracking and reporting of false defaulters. Lack of inter-operability between different electronic strategic information platforms. Planned case-based MTCT tracking mechanisms (pregnant women & new infant infections). Plans to develop and roll-out unique identifier code (UIC)"  *Zimbabwe National HIV and AIDS Strategic Plan 2021–2025. National AIDS Council; 2020, pages 58 and 81* | Fully implemented | Ongoing | Routine data |
| Key population surveys (size and prevalence) | Yes  Population size estimates of key populations presented.  *Zimbabwe National HIV and AIDS Strategic Plan 2021–2025. National AIDS Council; 2020, page 28* | Fully implemented | Not stated | Surveys |
| HIV ART Care records (Number on ART, ART outcomes) | Yes  ART coverage estimates presented  Zimbabwe National HIV and AIDS Strategic Plan 2021–2025. National AIDS Council; 2020, page 49 | Fully implemented | Ongoing | Routine data |
| HIV drug resistance surveys | Not mentioned  Other sources report drug resistance surveys linked to PHIA surveys | Fully implemented | Not stated | Surveys |
| Pharmacovigilance (e.g. birth defects surveillance) | Not stated  But other research shows pharmacovigilance activities: doi: [10.1371/journal.pone.0200459](https://doi.org/10.1371/journal.pone.0200459) | Partially implemented | Not stated | Surveys |
| HIV-associated infections (e.g Cervical cancer, TB, Hepatitis) | Yes  Stated on pages 55 to 58  Zimbabwe National HIV and AIDS Strategic Plan 2021–2025. National AIDS Council; 2020 | Fully implemented | Ongoing | Routine data |
| HIV prevalence in blood donors | Yes  HIV screening of blood mentioned.  Zimbabwe National HIV and AIDS Strategic Plan 2021–2025. National AIDS Council; 2020, page 90 | Fully implemented | Ongoing | Routine data |
| **Mortality** |  |  |  |  |
| Mathematical modelling | No  But modelling estimates of mortality presented on pages 15 and 16 | Fully implemented | Annually | Modelling |
| Vital registration (national, DHSS) | No | Not documented | Not documented | NA |
| Community mortality reporting systems/surveys | No | Not documented | Not documented | NA |
| Facility-based mortality reporting systems | No | Not documented | Not documented | NA |

Abbreviations: DHS=Demographic and Health survey, MICS=Multiple Indicator Survey, STI=Sexually transmitted infections, AGYW=Adolescent Girls and Young Women, ANC=Antenatal Clinic, PMTCT=Prevention of mother to child transmission, LIMS=Laboratory information management system, NHLS=National health laboratory System, DHSS=Demographic health surveillance site, NA= information not available
